# Machine Learning for the Diagnosis of Parkinson's Disease: A Review of Literature

**DOI:** 10.3389/fnagi.2021.633752

**Published:** 2021-05-06

**Authors:** Jie Mei, Christian Desrosiers, Johannes Frasnelli

**Affiliations:** ^1^Chemosensory Neuroanatomy Lab, Department of Anatomy, Université du Québec à Trois-Rivières (UQTR), Trois-Rivières, QC, Canada; ^2^Laboratoire d'Imagerie, de Vision et d'Intelligence Artificielle (LIVIA), Department of Software and IT Engineering, École de Technologie Supérieure, Montreal, QC, Canada; ^3^Centre de Recherche de l'Hôpital du Sacré-Coeur de Montréal, Centre Intégré Universitaire de Santé et de Services Sociaux du Nord-de-l'Île-de-Montréal (CIUSSS du Nord-de-l'Île-de-Montréal), Montreal, QC, Canada

**Keywords:** Parkinson's disease, machine learning, deep learning, diagnosis, differential diagnosis

## Abstract

Diagnosis of Parkinson's disease (PD) is commonly based on medical observations and assessment of clinical signs, including the characterization of a variety of motor symptoms. However, traditional diagnostic approaches may suffer from subjectivity as they rely on the evaluation of movements that are sometimes subtle to human eyes and therefore difficult to classify, leading to possible misclassification. In the meantime, early non-motor symptoms of PD may be mild and can be caused by many other conditions. Therefore, these symptoms are often overlooked, making diagnosis of PD at an early stage challenging. To address these difficulties and to refine the diagnosis and assessment procedures of PD, machine learning methods have been implemented for the classification of PD and healthy controls or patients with similar clinical presentations (e.g., movement disorders or other Parkinsonian syndromes). To provide a comprehensive overview of data modalities and machine learning methods that have been used in the diagnosis and differential diagnosis of PD, in this study, we conducted a literature review of studies published until February 14, 2020, using the PubMed and IEEE Xplore databases. A total of 209 studies were included, extracted for relevant information and presented in this review, with an investigation of their aims, sources of data, types of data, machine learning methods and associated outcomes. These studies demonstrate a high potential for adaptation of machine learning methods and novel biomarkers in clinical decision making, leading to increasingly systematic, informed diagnosis of PD.

## Introduction

Parkinson's disease (PD) is one of the most common neurodegenerative diseases with a prevalence rate of 1% in the population above 60 years old, affecting 1–2 people per 1,000 (Tysnes and Storstein, 2017). The estimated global population affected by PD has more than doubled from 1990 to 2016 (from 2.5 million to 6.1 million), which is a result of increased number of elderly people and age-standardized prevalence rates (Dorsey et al., 2018). PD is a progressive neurological disorder associated with motor and non-motor features (Jankovic, 2008) which comprises multiple aspects of movements, including planning, initiation and execution (Contreras-Vidal and Stelmach, 1995).

During its development, movement-related symptoms such as tremor, rigidity and difficulties in initiation can be observed, prior to cognitive and behavioral alterations including dementia (Opara et al., 2012). PD severely affects patients' quality of life (QoL), social functions and family relationships, and places heavy economic burdens at individual and society levels (Johnson et al., 2013; Kowal et al., 2013; Yang and Chen, 2017).

The diagnosis of PD is traditionally based on motor symptoms. Despite the establishment of cardinal signs of PD in clinical assessments, most of the rating scales used in the evaluation of disease severity have not been fully evaluated and validated (Jankovic, 2008). Although non-motor symptoms (e.g., cognitive changes such as problems with attention and planning, sleep disorders, sensory abnormalities such as olfactory dysfunction) are present in many patients prior to the onset of PD (Jankovic, 2008; Tremblay et al., 2017), they lack specificity, are complicated to assess and/or yield variability from patient to patient (Zesiewicz et al., 2006). Therefore, non-motor symptoms do not yet allow for diagnosis of PD independently (Braak et al., 2003), although some have been used as supportive diagnostic criteria (Postuma et al., 2015).

Machine learning techniques are being increasingly applied in the healthcare sector. As its name implies, machine learning allows for a computer program to learn and extract meaningful representation from data in a semi-automatic manner. For the diagnosis of PD, machine learning models have been applied to a multitude of data modalities, including handwritten patterns (Drotár et al., 2015; Pereira et al., 2018), movement (Yang et al., 2009; Wahid et al., 2015; Pham and Yan, 2018), neuroimaging (Cherubini et al., 2014a; Choi et al., 2017; Segovia et al., 2019), voice (Sakar et al., 2013; Ma et al., 2014), cerebrospinal fluid (CSF) (Lewitt et al., 2013; Maass et al., 2020), cardiac scintigraphy (Nuvoli et al., 2019), serum (Váradi et al., 2019), and optical coherence tomography (OCT) (Nunes et al., 2019). Machine learning also allows for combining different modalities, such as magnetic resonance imaging (MRI) and single-photon emission computed tomography (SPECT) data (Cherubini et al., 2014b; Wang et al., 2017), in the diagnosis of PD. By using machine learning approaches, we may therefore identify relevant features that are not traditionally used in the clinical diagnosis of PD and rely on these alternative measures to detect PD in preclinical stages or atypical forms.

In recent years, the number of publications on the application of machine learning to the diagnosis of PD has increased. Although previous studies have reviewed the use of machine learning in the diagnosis and assessment of PD, they were limited to the analysis of motor symptoms, kinematics, and wearable sensor data (Ahlrichs and Lawo, 2013; Ramdhani et al., 2018; Belić et al., 2019). Moreover, some of these reviews only included studies published between 2015 and 2016 (Pereira et al., 2019). In this study, we aim to (a) comprehensively summarize all published studies that applied machine learning models to the diagnosis of PD for an exhaustive overview of data sources, data types, machine learning models, and associated outcomes, (b) assess and compare the feasibility and efficiency of different machine learning methods in the diagnosis of PD, and (c) provide machine learning practitioners interested in the diagnosis of PD with an overview of previously used models and data modalities and the associated outcomes, and recommendations on how experimental protocols and results could be reported to facilitate reproduction. As a result, the application of machine learning to clinical and non-clinical data of different modalities has often led to high diagnostic accuracies in human participants, therefore may encourage the adaptation of machine learning algorithms and novel biomarkers in clinical settings to assist more accurate and informed decision making.

## Methods

### Search Strategy

A literature search was conducted on the PubMed (https://pubmed.ncbi.nlm.nih.gov) and IEEE *Xplore* (https://ieeexplore.ieee.org/search/advanced/command) databases on February 14, 2020 for all returned results. Boolean search strings used are shown in Table 1. No additional filters were applied in the literature search. All retrieved studies were systematically identified, screened and extracted for relevant information following the Preferred Reporting Items for Systematic Reviews and Meta-Analyses (PRISMA) guidelines (Moher et al., 2009).

**Table 1:** Boolean search strings used for the retrieval of relevant publications on PubMed and IEEE Xplore databases.

**Database**	**Boolean search string**
PubMed	(“Parkinson Disease”[Mesh] OR Parkinson*) AND (“Machine Learning”[Mesh] OR machine learn* OR machine-learn* OR deep learn* OR deep-learn*) AND (human OR patient) AND (“Diagnosis”[Mesh] OR diagnos* OR detect* OR classif* OR identif*) NOT review[Publication Type]
IEEE *Xplore*	(Parkinson*) AND (machine learn* OR machine-learn* OR deep learn* OR deep-learn*) AND (human OR patient) AND (diagnosis OR diagnose OR diagnosing OR detection OR detect OR detecting OR classification OR classify OR classifying OR identification OR identify OR identifying)

### Inclusion and Exclusion Criteria

Studies that satisfy one or more of the following criteria and used machine learning methods were included:

1. Classification of PD from healthy controls (HC),
2. Classification of PD from Parkinsonism (e.g., progressive supranuclear palsy (PSP) and multiple system atrophy (MSA)), and
3. Classification of PD from other movement disorders (e.g., essential tremor (ET)).

Studies falling into one or more of the following categories were excluded:

1. Studies related to Parkinsonism or/and diseases other than PD that did not involve classification or detection of PD (e.g., differential diagnosis of PSP, MSA, and other atypical Parkinsonian disorders),
2. Studies not related to the diagnosis of PD (e.g., subtyping or severity assessment, analysis of behavior, disease progression, treatment outcome prediction, identification, and localization of brain structures or parameter optimization during surgery),
3. Studies related to the diagnosis of PD, but performed analysis and assessed model performance at sample level (e.g., classification using individual MRI scans without aggregating scan-level performance to patient level),
4. Classification of PD from non-Parkinsonism (e.g., Alzheimer's disease),
5. Study did not use metrics that measure classification performance,
6. Study used organisms other than human (e.g., *Caenorhabditis elegans*, mice or rats),
7. Study did not provide sufficient or accurate descriptions of machine learning methods, datasets or subjects used (e.g., does not provide sample size, or incorrectly described the dataset(s) used),
8. Not original journal article or conference proceedings papers (e.g., review and viewpoint paper), and
9. In languages other than English.

### Data Extraction

The following information is included in the data extraction table: (1) objectives, (2) type of diagnosis (diagnosis, differential diagnosis, sub-typing), (3) data source, (4) data type, (5) number of subjects, (6) machine learning method(s), splitting strategy and cross validation, (7) associated outcomes, (8) year, and (9) reference.

For studies published online first and archived in another year, “year of publication” was defined as the year during which the study was published online. If this information was unavailable, the year in which the article was copyrighted was regarded as the year of publication. For studies that introduced novel models and used existing models merely for comparison, information related to the novel models was extracted. Classification of PD and scans without evidence for dopaminergic deficit (SWEDD) was treated as subtyping (Erro et al., 2016).

### Study Objectives

To outline the different goals and objectives of included studies, we have further categorized them based on the type of diagnosis and their general aim. From the perspective of diagnostics, these studies could be divided into (a) the diagnosis or detection of PD (which compares data collected from PD patients and healthy controls), (b) differential diagnosis (discrimination between patients with idiopathic PD and patients with atypical Parkinsonism), and (c) sub-typing (discrimination among sub-types of PD).

Included studies were also analyzed for their general aim: For studies with a focus on the development of novel technical approaches to be used in the diagnosis of Parkinson's disease, e.g., new machine learning and deep learning models and architectures, data acquisition devices, and feature extraction algorithms that haven't been previously presented and/or employed, we defined them as (a) “*methodology*” studies. Studies that validate and investigate (a) the application of previously published and validated machine learning and deep learning models, and/or (b) the feasibility of introducing data modalities that are not commonly used in the machine learning-based diagnosis of PD (e.g., CSF data), were defined as (b) “*clinical application*” studies.

### Model Evaluation

In the present study, accuracy was used to compare performance of machine learning models. For each data type, we summarized the type of machine learning models that led to the per-study highest accuracy. However, in some studies, only one machine learning model was tested. Therefore, we define “model associated with the per-study highest accuracy” as (a) the only model implemented and used in a study or (b) the model that achieved the highest accuracy or that was highlighted in studies that used multiple models. Results are expressed as mean (SD).

For studies reporting both training and testing/validation accuracy, testing or validation accuracy was considered. For studies that reported both validation and test accuracy, test accuracy was considered. For studies with more than one dataset or classification problem (e.g., HC vs. PD and HC vs. idiopathic hyposmia vs. PD), accuracy was averaged across datasets or classification problems. For studies that reported classification accuracy for each group of subjects individually, accuracy was averaged across groups. For studies reporting a range of accuracies or accuracies given by different cross validation methods or feature combinations, the highest accuracies were considered. In studies that compared HC with diseases other than PD or PD with diseases other than Parkinsonism, diagnosis of diseases other than PD or Parkinsonism (e.g., amyotrophic lateral sclerosis) was not considered. Accuracy of severity assessment was not considered.

## Results

### Literature Review

**Figure 1:**
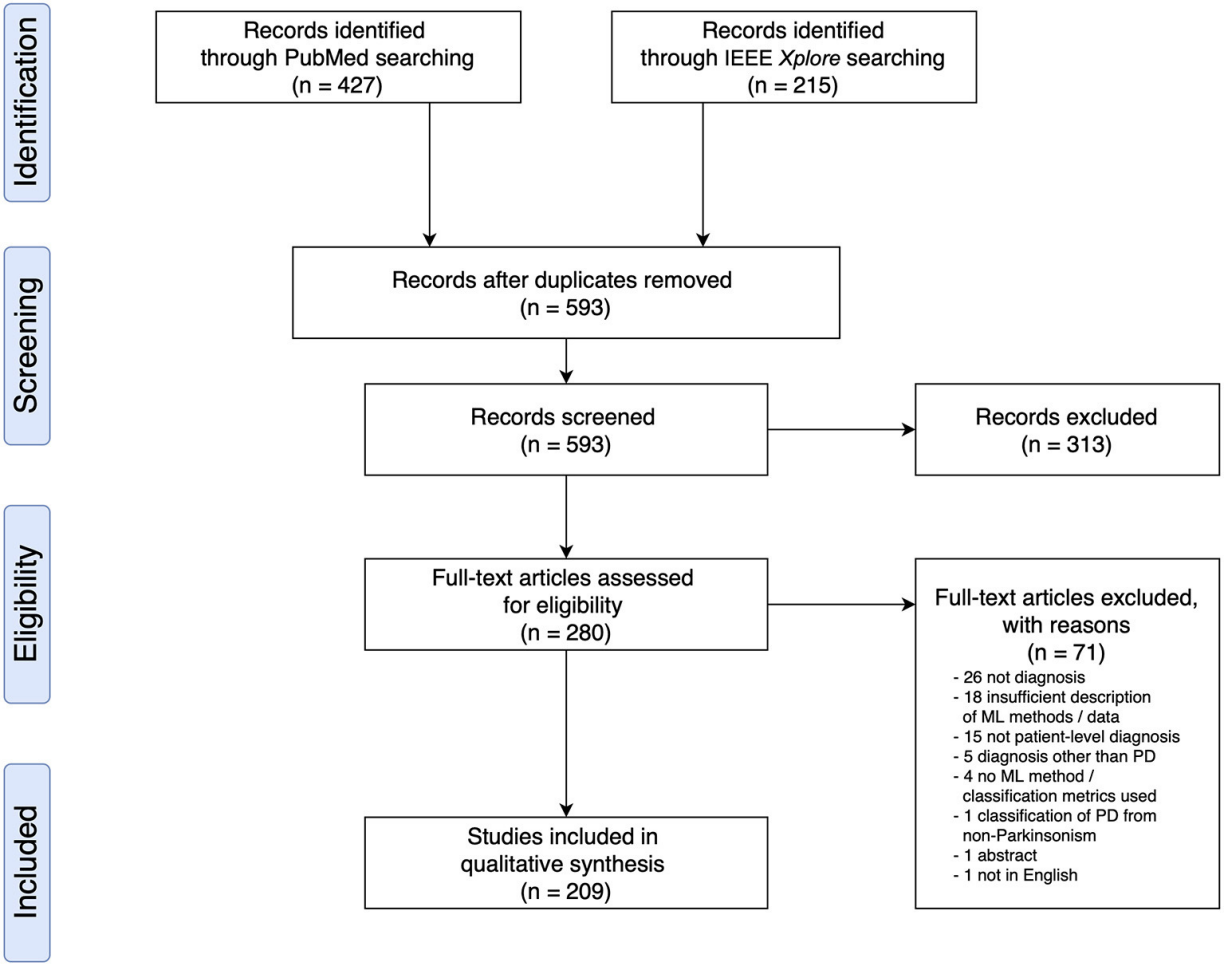
PRISMA Flow Diagram of Literature Search and Selection Process showing the number of studies identified, screened, extracted, and included in the review.

Based on the search criteria, we retrieved 427 (PubMed) and 215 (IEEEXplore) search results, leading to a total of 642 publications. After removing duplicates, we screened 593 publications for titles and abstracts, following which we excluded 313 based on the exclusion criteria and examined 280 full text articles. Overall, we included 209 research articles for data extraction (Figure 1 and see Supplementary Materials for a full list of included studies). All articles were published from the year 2009 onwards, and an increase in the number of papers published per year was observed (Supplementary Figure 1).

### Data Source and Sample Size

In 93 out of 209 studies (43.1%), original data were collected from human participants. In 108 studies (51.7%), data used were from public repositories and databases, including University of California at Irvine (UCI) Machine Learning Repository (Dua and Graff, 2018) (*n* = 44), Parkinson's Progression Markers Initiative (Marek et al., 2011) (PPMI; *n* = 33), PhysioNet (Goldberger et al., 2000) (*n* = 15), HandPD dataset (Pereira et al., 2015) (*n* = 6), mPower database (Bot et al., 2016) (*n* = 4), and 6 other databases (Mucha et al., 2018; Vlachostergiou et al., 2018; Bhati et al., 2019; Hsu et al., 2019; Taleb et al., 2019; Wodzinski et al., 2019; Table 2).

**Table 2:** Source of data of the included studies.

**Data source/Database**	**Number of studies**	**Percentage**
independent recruitment of human participants	93	43.06%
UCI Machine Learning Repository	44	20.37%
PPMI database	33	15.28%
PhysioNet	15	6.94%
HandPD dataset	6	2.78%
mPower database	4	1.85%
Other databases (1 PACS, 1 PaHaW, 1 PC-GITA database, 1 PDMultiMC database, 1 Neurovoz corpus, 1 The NTUA Parkinson Dataset)	6	2.78%
Collected postmortem	1	0.46%
Commercially sourced	1	0.46%
Acquired at another institution	1	0.46%
From another study	1	0.46%
From the author's institutional database	1	0.46%
Others (1 PPMI + Sheffield Teaching Hospitals NHS Foundation Trust; 1 PPMI + Seoul National University Hospital cohort; 1 UCI + collected from participants)	3	1.39%

*PACS, Picture Archiving and Communication System; PaHaW, Parkinson's Disease Handwriting Database*.

In 3 studies, data from public repositories were combined with data from local databases or participants (Agarwal et al., 2016; Choi et al., 2017; Taylor and Fenner, 2017). In the remaining studies, data were sourced (Wahid et al., 2015) from another study (Fernandez et al., 2013), collected at another institution (Segovia et al., 2019), obtained from the authors' institutional database (Nunes et al., 2019), collected postmortem (Lewitt et al., 2013), or commercially sourced (Váradi et al., 2019).

**Figure 2:**
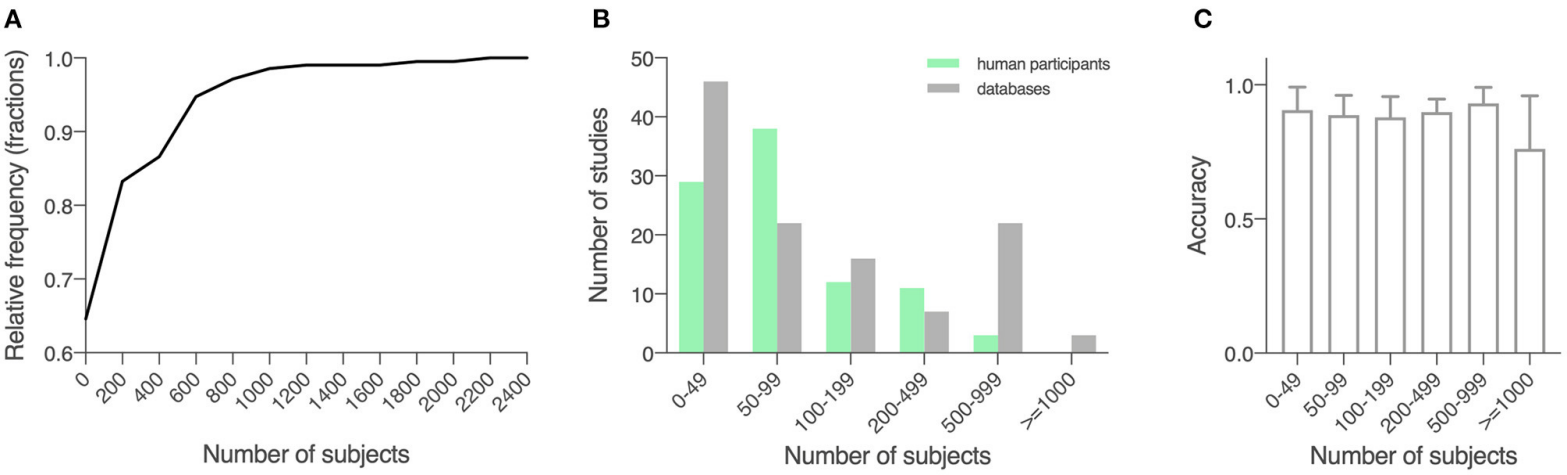
Sample size of the included studies. **(A)** Cumulative relative frequency graph depicting the frequency of the sample sizes studied. **(B)** Histogram depicting the frequency of a sample size of 0–50, 50–100, 100–200, 200–500, 500–100, and over 1,000 for studies using locally recruited human participants and studies using previously published open databases. Green, studies using locally recruited human participants; gray, studies using data sourced from public databases. **(C)** Model performance as measured by accuracy in relation to sample size, shown in means (SD).

The 209 studies had an average sample size of 184.6 (289.3), with a smallest sample size of 10 (Kugler et al., 2013), and a largest sample size of 2,289 (Tracy et al., 2019; Figure 2A). For studies that recruited human participants (*n* = 93), data from an average of 118.0 (142.9) participants were collected (range: 10–920; Figure 2B). For other studies (*n* = 116), an average sample size of 238.1 (358.5) was reported (range: 30–2,289; Figure 2B). For a description of average accuracy reported in these studies in relation to sample size, see Figure 2C.

### Study Objectives

In included studies, although “diagnosis of PD” was used as the search criteria, machine learning had been applied for diagnosis (PD vs. HC), differential diagnosis (idiopathic PD vs. atypical Parkinsonism) and sub-typing (differentiation of sub-types of PD) purposes. Most studies focused on diagnosis (*n* = 168, 80.4%) or differential diagnosis (*n* = 20, 9.6%). Fourteen studies performed both diagnosis and differential diagnosis (6.7%), 5 studies (2.4%) diagnosed and subtyped PD, 2 studies (1.0%) included diagnosis, differential diagnosis, and subtyping.

Among the included studies, a total of 132 studies (63.2%) implemented and tested a machine learning method, a model architecture, a diagnostic system, a feature extraction algorithm, or a device for non-invasive, low-cost data acquisition that hasn't been established for the detection and early diagnosis of PD (*methodology* studies). In 77 studies (36.8%), previously proposed and validated machine learning methods were tested in clinical settings for early detection of PD, identification of novel biomarkers or examination of uncommonly used data modalities for the diagnosis of PD (e.g., CSF; *clinical application* studies).

### Comparing Studies With Different Objectives

#### Source of Data

In the 132 studies that proposed or tested novel machine learning methods (i.e., *methodology* studies), a majority used data from publicly available databases (*n* = 89, 67.4%). Data collected from human participants were used in 41 studies (31.1%) and the two remaining studies (1.5%) used commercially sourced data or data from both existing public databases and local participants specifically recruited for the study. Out of the 77 studies that used machine learning models in clinical settings (i.e., *clinical application* studies), 52 (67.5%) collected data from human participants, 22 (28.6%) used data from public databases. Two (2.6%) studies obtained data from a database and a local cohort, and 1 (1.3%) study collected data postmortem.

#### Data Modality

**Figure 3:**
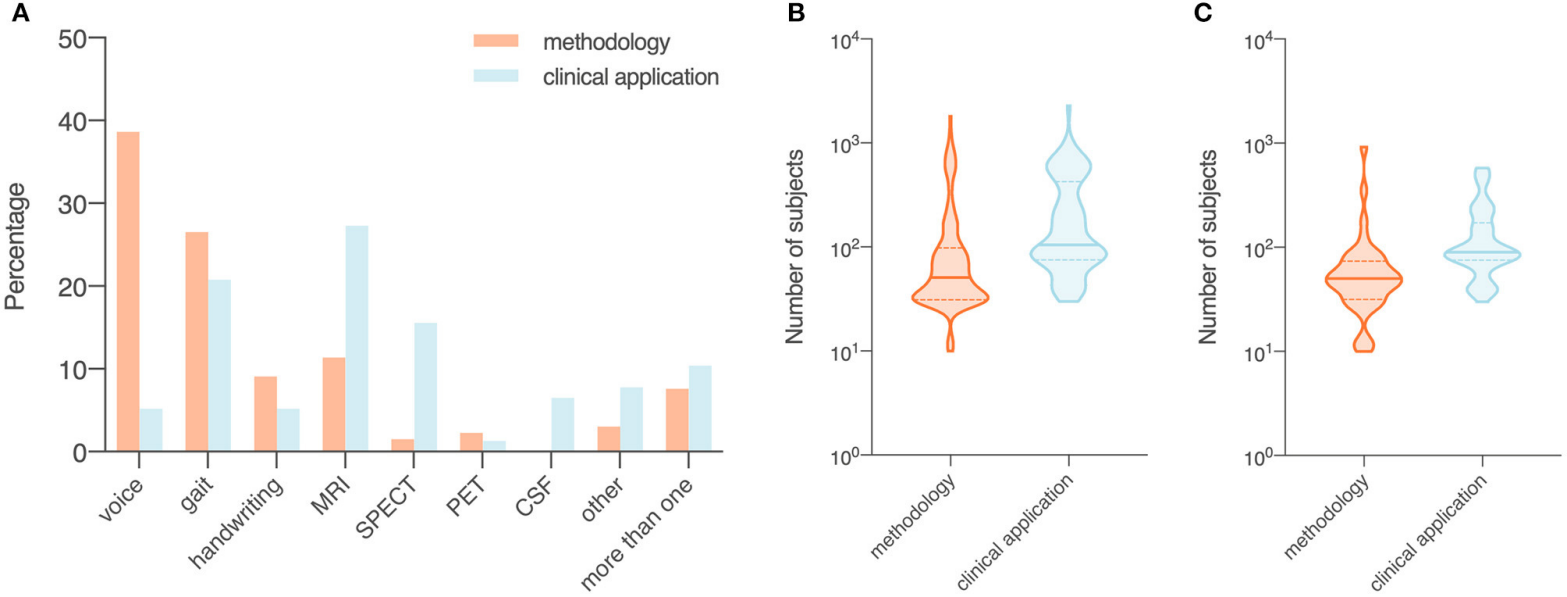
Data modality **(A)** and number of subjects **(B,C)** of included studies, summarized by objectives (i.e., *methodology* or *clinical application*). Orange, studies with a focus on the development of a novel technical approach to be used in the diagnosis of Parkinson's disease (i.e., *methodology*); blue, studies that investigate the use of published machine learning models or novel data modalities (i.e., *clinical application*). **(A)** Proportion of data modalities in included studies displayed as percentages. **(B)** Sample size in all included studies. **(C)** Sample size in studies that collected data from recruited human participants. Data shown are means (SD).

In *methodology* studies, the most commonly used data modalities were voice recordings (*n* = 51, 38.6%), movement (*n* = 35, 26.5%), and MRI data (*n* = 15, 11.4%). For studies on *clinical applications*, MRI data (*n* = 21, 27.3%), movement (*n* = 16, 20.8%), and SPECT imaging data (*n* = 12, 15.6%) were of high relevance. All studies using CSF features (*n* = 5) focused on validation of existing machine learning models in a clinical setting (Figure 3A).

#### Number of Subjects

The average sample size was 137.1 for the 132 *methodology* studies (Figure 3B). For 41 out of the 132 studies that used data from recruited human participants, the average sample size was 81.7 (Figure 3C). In the 77 studies on *clinical applications*, the average sample size was 266.2 (Figure 3B). For 52 out of the 77 clinical studies that collected data from recruited participants, the average sample size was 145.9 (Figure 3C).

### Machine Learning Methods Applied to the Diagnosis of PD

We divided 448 machine learning models from the 209 studies into 8 categories: (1) support vector machine (SVM) and variants (*n* = 132 from 130 studies), (2) neural networks (*n* = 76 from 62 studies), (3) ensemble learning (*n* = 82 from 57 studies), (4) nearest neighbor and variants (*n* = 33 from 33 studies), (5) regression (*n* = 31 from 31 studies), (6) decision tree (*n* = 28 from 27 studies), (7) naïve Bayes (*n* = 26, from 26 studies), and (8) discriminant analysis (*n* = 12 from 12 studies). A small percentage of models used did not fall into any of the categories (*n* = 28, used in 24 studies).

On average, 2.14 machine learning models per study were applied to the diagnosis of PD. One study may have used more than one category of models. For a full description of data types used to train each type of machine learning models and the associated outcomes, see Supplementary Materials and Supplementary Figure 2.

### Performance Metrics

Various metrics have been used to assess the performance of machine learning models (Table 3). The most common metric was accuracy (*n* = 174, 83.3%), which was used individually (*n* = 55) or in combination with other metrics (*n* = 119) in model evaluation. Among the 174 studies that used accuracy, some have combined accuracy with sensitivity (i.e., recall) and specificity (*n* = 42), or with sensitivity, specificity and AUC (*n* = 16), or with recall (i.e., sensitivity), precision and F1 score (*n* = 7) for a more systematic understanding of model performance. A total of 35 studies (16.7%) used metrics other than accuracy. In these studies, the most used performance metrics were AUC (*n* = 19), sensitivity (*n* = 17), and specificity (*n* = 14), and the three were often applied together (*n* = 9) with or without other metrics.

**Table 3:** Performance metrics used in the evaluation of machine learning models.

**Performance metric**	**Definition**	**Number of studies**
Accuracy	$\frac{TP+TN}{TP+TN+FP+FN}$	174
Sensitivity (recall)	$\frac{TP}{TP+FN}$	110
Specificity (TNR)	$\frac{TN}{TN+FP}$	94
AUC	The two-dimensional area under the Receiver Operating Characteristic (ROC) curve	60
MCC	$\frac{TP\times TN-FP\times FN}{\sqrt{\left(TP+FP\right)\left(TP+FN\right)\left(TN+FP\right)\left(TN+FN\right)}}$	9
Precision (PPV)	$\frac{TP}{TP+FP}$	31
NPV	$\frac{TN}{TN+FN}$	8
F1 score	$2\times \;\frac{precision\times recall}{precision+recall}$	25
Others (7 kappa; 4 error rate; 3 EER; 1 MSE; 1 LOR; 1 confusion matrix; 1 cross validation score; 1 YI; 1 FPR; 1 FNR; 1 G-mean; 1 PE; 5 combination of metrics)	N/A	28

*TNR, true negative rate; AUC, Area under the ROC Curve; MCC, Matthews correlation coefficient; PPV, positive predictive value; NPV, negative predictive value; EER, equal error rate; MSE, mean squared error; LOR, log odds ratio; YI, Youden's Index; FPR, false positive rate; FNR, false negative rate; PE, probability excess*.

### Data Types and Associated Outcomes

Out of 209 studies, 122 (58.4%) applied machine learning methods to movement-related data, i.e., voice recordings (*n* = 55, 26.3%), movement data (*n* = 51, 24.4%), or handwritten patterns (*n* = 16, 7.7%). Imaging modalities analyzed including MRI (*n* = 36, 17.2%), SPECT (*n* = 14, 6.7%), and positron emission tomography (PET; *n* = 4, 1.9%). Five studies analyzed CSF samples (2.4%). In 18 studies (8.6%), a combination of different types of data was used.

Ten studies (4.8%) used data that do not belong to any categories mentioned above, such as single nucleotide polymorphisms (Cibulka et al., 2019) (SNPs), electromyography (EMG) (Kugler et al., 2013), OCT (Nunes et al., 2019), cardiac scintigraphy (Nuvoli et al., 2019), Patient Questionnaire of Movement Disorder Society Unified Parkinson's Disease Rating Scale (MDS-UPDRS) (Prashanth and Dutta Roy, 2018), whole-blood gene expression profiles (Shamir et al., 2017), transcranial sonography (Shi et al., 2018) (TCS), eye movements (Tseng et al., 2013), electroencephalography (EEG) (Vanegas et al., 2018), and serum samples (Váradi et al., 2019).

Given that studies used different data modalities and sources, and sometimes different samples of the same database, a summary of model performance, instead of direct comparison across studies, is provided.

#### Voice Recordings (*n* = 55)

**Figure 4:**
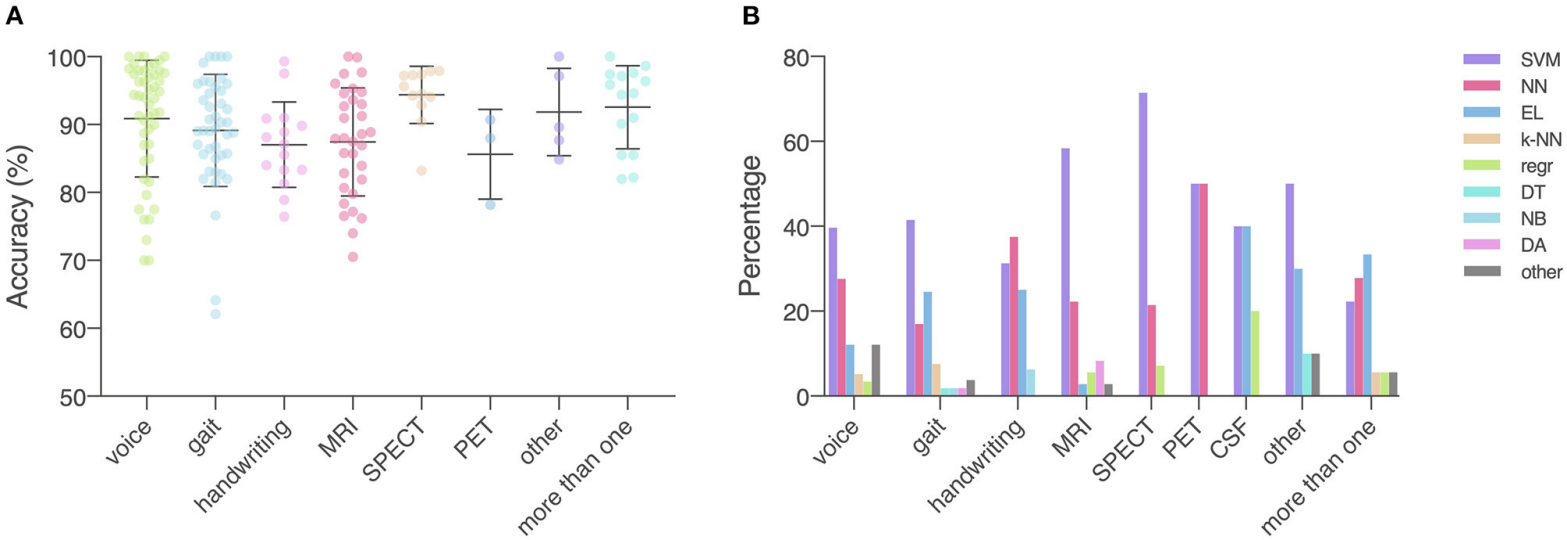
Data type, machine learning models applied, and accuracy. **(A)** Accuracy achieved in individual studies and average accuracy for each data type. Error bar: standard deviation. **(B)** Distribution of machine learning models applied per data type. MRI, magnetic resonance imaging; SPECT, single-photon emission computed tomography; PET, positron emission tomography; CSF, cerebrospinal fluid; SVM, support vector machine; NN, neural network; EL, ensemble learning; k-NN, nearest neighbor; regr, regression; DT, decision tree; NB, naïve Bayes; DA, discriminant analysis; other: data/models that do not belong to any of the given categories.

The 49 studies that used accuracy to evaluate machine learning models achieved an average accuracy of 90.9 (8.6) % (Figure 4A), ranging from 70.0% (Kraipeerapun and Amornsamankul, 2015; Ali et al., 2019a) to 100.0% (Hariharan et al., 2014; Abiyev and Abizade, 2016; Ali et al., 2019c; Dastjerd et al., 2019). In 3 studies, the highest accuracy was achieved by two types of machine learning models individually, namely regression or SVM (Ali et al., 2019a), neural network or SVM (Hariharan et al., 2014), and ensemble learning or SVM (Mandal and Sairam, 2013). The per-study highest accuracy was achieved with SVM in 23 studies (39.7%), with neural network in 16 studies (27.6%), with ensemble learning in 7 studies (12.1%), with nearest neighbor in 3 studies (5.2%), and with regression in 2 studies (3.4%). Models that do not belong to any given categories led to the per-study highest accuracy in 7 studies (12.1%; Figure 4B).

Voice recordings from the UCI machine learning repository were used in 42 studies (Table 4). Among the 42 studies, 39 used accuracy to evaluate classification performance and the average accuracy was 92.0 (9.0) %. The lowest accuracy was 70.0% and the highest accuracy was 100.0%. Eight out of 9 studies that collected voice recordings from human participants used accuracy as the performance metric, and the average, lowest and highest accuracies were 87.7 (6.8) %, 77.5%, and 98.6%, respectively. The 4 remaining studies used data from the Neurovoz corpus (*n* = 1), mPower database (*n* = 1), PC-GITA database (*n* = 1), or data from both the UCI machine learning repository and human participants (*n* = 1). Two out of these 4 studies used accuracy to evaluate model performance and reported an accuracy of 81.6 and 91.7%.

**Table 4:** Studies that applied machine learning models to voice recordings to diagnose PD (*n* = 55).

**Objectives**	**Type of diagnosis**	**Source of data**	**Number of subjects (*n*)**	**Machine learning method(s), splitting strategy and cross validation**	**Outcomes**	**Year**	**References**
Classification of PD from HC	Diagnosis	UCI machine learning repository	31; 8 HC + 23 PD	Fuzzy neural system with 10-fold cross validation	Testing accuracy = 100%	2016	Abiyev and Abizade, 2016
Classification of PD from HC	Diagnosis	UCI machine learning repository	31; 8 HC + 23 PD	RPART, C4.5, PART, Bagging CART, random forest, Boosted C5.0, SVM	SVM:	2019	Aich et al., 2019
					Accuracy = 97.57%		
					Sensitivity = 0.9756		
					Specificity = 0.9987		
					NPV = 0.9995		
Classification of PD from HC	Diagnosis	UCI machine learning repository	31; 8 HC + 23 PD	DBN of 2 RBMs	Testing accuracy = 94%	2016	Al-Fatlawi et al., 2016
Classification of PD from HC	Diagnosis	UCI machine learning repository	31; 8 HC + 23 PD	EFMM-OneR with 10-fold cross validation or 5-fold cross validation	Accuracy = 94.21%	2019	Sayaydeha and Mohammad, 2019
Classification of PD from HC	Diagnosis	UCI machine learning repository	40; 20 HC + 20 PD	Linear regression, LDA, Gaussian naïve Bayes, decision tree, KNN, SVM-linear, SVM-RBF with leave-one-subject-out cross validation	Logistic regression or SVM-linear accuracy = 70%	2019	Ali et al., 2019a
Classification of PD from HC	Diagnosis	UCI machine learning repository	40; 20 HC + 20 PD	LDA-NN-GA with leave-one-subject-out cross validation	Training:	2019	Ali et al., 2019c
					Accuracy = 95%		
					Sensitivity = 95%		
					Test:		
					Accuracy = 100%		
					Sensitivity = 100%		
Classification of PD from HC	Diagnosis	UCI machine learning repository	31; 8 HC + 23 PD	NNge with AdaBoost with 10-fold cross validation	Accuracy = 96.30%	2018	Alqahtani et al., 2018
Classification of PD from HC	Diagnosis	UCI machine learning repository	31; 8 HC + 23 PD	Logistic regression, KNN, naïve Bayes, SVM, decision tree, random forest, DNN with 10-fold cross validation	KNN accuracy = 95.513%	2018	Anand et al., 2018
Classification of PD from HC	Diagnosis	UCI machine learning repository	31; 8 HC + 23 PD	MLP with a train-validation-test ratio of 50:20:30	Training accuracy = 97.86%	2012	Bakar et al., 2012
					Test accuracy = 92.96%		
					MSE = 0.03552		
Classification of PD from HC	Diagnosis	UCI machine learning repository	31 (8 HC + 23 PD) for dataset 1 and 68 (20 HC + 48 PD) for dataset 2	FKNN, SVM, KELM with 10-fold cross validation	FKNN accuracy = 97.89%	2018	Cai et al., 2018
Classification of PD from HC	Diagnosis	UCI machine learning repository	40; 20 HC + 20 PD	SVM, logistic regression, ET, gradient boosting, random forest with train-test split ratio = 80:20	Logistic regression accuracy = 76.03%	2019	Celik and Omurca, 2019
Classification of PD from HC	Diagnosis	UCI machine learning repository	40; 20 HC + 20 PD	MLP, GRNN with a training-test ratio of 50:50	GRNN:	2016	Çimen and Bolat, 2016
					Error rate = 0.0995 (spread parameter = 195.1189)		
					Error rate = 0.0958 (spread parameter = 1.2)		
					Error rate = 0.0928 (spread parameter = 364.8)		
Classification of PD from HC	Diagnosis	UCI machine learning repository	31; 8 HC + 23 PD	ECFA-SVM with 10-fold cross validation	Accuracy = 97.95%	2017	Dash et al., 2017
					Sensitivity = 97.90%		
					Precision = 97.90%		
					F-measure = 97.90%		
					Specificity = 96.50%		
					AUC = 97.20%		
Classification of PD from HC	Diagnosis	UCI machine learning repository	40; 20 HC + 20 PD	Fuzzy classifier with 10-fold cross validation, leave-one-out cross validation or a train-test ratio of 70:30	Accuracy = 100%	2019	Dastjerd et al., 2019
Classification of PD from HC	Diagnosis	UCI machine learning repository	31; 8 HC + 23 PD	Averaged perceptron, BPM, boosted decision tree, decision forests, decision jungle, locally deep SVM, logistic regression, NN, SVM with 10-fold cross-validation	Boosted decision trees:	2017	Dinesh and He, 2017
					Accuracy = 0.912105		
					Precision = 0.935714		
					F-score = 0.942368		
					AUC = 0.966293		
Classification of PD from HC	Diagnosis	UCI machine learning repository	50; 8 HC + 42 PD	KNN, SVM, ELM with a train-validation ratio of 70:30	SVM:	2017	Erdogdu Sakar et al., 2017
					Accuracy = 96.43%		
					MCC = 0.77		
Classification of PD from HC	Diagnosis	UCI machine learning repository	252; 64 HC + 188 PD	CNN with leave-one-person-out cross validation	Accuracy = 0.869	2019	Gunduz, 2019
					F-measure = 0.917		
					MCC = 0.632		
Classification of PD from HC	Diagnosis	UCI machine learning repository	31; 8 HC + 23 PD	SVM, logistic regression, KNN, DNN with a train-test ratio of 70:30	DNN:	2018	Haq et al., 2018
					Accuracy = 98%		
					Specificity = 95%		
					sensitivity = 99%		
Classification of PD from HC	Diagnosis	UCI machine learning repository	31; 8 HC + 23 PD	SVM-RBF, SVM-linear with 10-fold cross validation	Accuracy = 99%	2019	Haq et al., 2019
					Specificity = 99%		
					Sensitivity = 100%		
Classification of PD from HC	Diagnosis	UCI machine learning repository	31; 8 HC + 23 PD	LS-SVM, PNN, GRNN with conventional (train-test ratio of 50:50) and 10-fold cross validation	LS-SVM or PNN or GRNN:	2014	Hariharan et al., 2014
					Accuracy = 100%		
					Precision = 100%		
					Sensitivity = 100%		
					specificity = 100%		
					AUC = 100		
Classification of PD from HC	Diagnosis	UCI machine learning repository	31; 8 HC + 23 PD	Random tree, SVM-linear, FBANN with 10-fold cross validation	FBANN:	2014	Islam et al., 2014
					Accuracy = 97.37%		
					Sensitivity = 98.60%		
					Specificity = 93.62%		
					FPR = 6.38%		
					Precision = 0.979		
					MSE = 0.027		
Classification of PD from HC	Diagnosis	UCI machine learning repository	31; 8 HC + 23 PD	SVM-linear with 5-fold cross validation	Error rate ~0.13	2012	Ji and Li, 2012
Classification of PD from HC	Diagnosis	UCI machine learning repository	40; 20 HC + 20 PD	Decision tree, random forest, SVM, GBM, XGBoost	SVM-linear:	2018	Junior et al., 2018
					FNR = 10%		
					Accuracy = 0.725		
Classification of PD from HC	Diagnosis	UCI machine learning repository	31; 8 HC + 23 PD	CART, SVM, ANN	SVM accuracy = 93.84%	2020	Karapinar Senturk, 2020
Classification of PD from HC	Diagnosis	UCI machine learning repository	Dataset 1: 31; 8 HC + 23 PD Dataset 2: 40; 20 HC + 20 PD	EWNN with a train-test ratio of 90:10 and cross validation	Dataset 1: Accuracy = 92.9%	2018	Khan et al., 2018
					Ensemble classification accuracy = 100.0%		
					Sensitivity = 100.0%		
					MCC = 100.0%		
					Dataset 2:		
					Accuracy = 66.3%		
					Ensemble classification accuracy = 90.0%		
					Sensitivity = 93.0%		
					Specificity = 97.0%		
					MCC = 87.0%		
Classification of PD from HC	Diagnosis	UCI machine learning repository	40; 20 HC + 20 PD	Stacked generalization with CMTNN with 10-fold cross validation	Accuracy = ~70%	2015	Kraipeerapun and Amornsamankul, 2015
Classification of PD from HC	Diagnosis	UCI machine learning repository	40; 20 HC + 20 PD	HMM, SVM	HMM:	2019	Kuresan et al., 2019
					Accuracy = 95.16%		
					Sensitivity = 93.55%		
					Specificity = 91.67%		
Classification of PD from HC	Diagnosis	UCI machine learning repository	31; 8 HC + 23 PD	IGWO-KELM with 10-fold cross validation	Iteration number = 100	2017	Li et al., 2017
					Accuracy = 97.45%		
					Sensitivity = 99.38%		
					Specificity = 93.48%		
					Precision = 97.33%		
					G-mean = 96.38%		
					F-measure = 98.34%		
Classification of PD from HC	Diagnosis	UCI machine learning repository	31; 8 HC + 23 PD	SCFW-KELM with 10-fold cross validation	Accuracy = 99.49%	2014	Ma et al., 2014
					Sensitivity = 100%		
					Specificity = 99.39%		
					AUC = 99.69%		
					F-measure = 0.9966		
					Kappa = 0.9863		
Classification of PD from HC	Diagnosis	UCI machine learning repository	31; 8 HC + 23 PD	SVM-RBF with 10-fold cross validation	Accuracy = 96.29%	2016	Ma et al., 2016
					Sensitivity = 95.00%		
					Specificity = 97.50%		
Classification of PD from HC	Diagnosis	UCI machine learning repository	31; 8 HC + 23 PD	Logistic regression, NN, SVM, SMO, Pegasos, AdaBoost, ensemble selection, FURIA, rotation forest Bayesian network with 10-fold cross-validation	Average accuracy across all models = 97.06% SMO, Pegasos, or AdaBoost accuracy = 98.24%	2013	Mandal and Sairam, 2013
Classification of PD from HC	Diagnosis	UCI machine learning repository	31; 8 HC + 23 PD	Logistic regression, KNN, SVM, naïve Bayes, decision tree, random forest, ANN	ANN:	2018	Marar et al., 2018
					Accuracy = 94.87%		
					Specificity = 96.55%		
					Sensitivity = 90%		
Classification of PD from HC	Diagnosis	UCI machine learning repository	Dataset 1: 31; 8 HC + 23 PD	KNN	Dataset 1 accuracy = 90%	2017	Moharkan et al., 2017
			Dataset 2: 40; 20 HC + 20 PD		Dataset 2 accuracy = 65%		
Classification of PD from HC	Diagnosis	UCI machine learning repository	31; 8 HC + 23 PD	Rotation forest ensemble with 10-fold cross validation	Accuracy = 87.1%	2011	Ozcift and Gulten, 2011
					Kappa error = 0.63		
					AUC = 0.860		
Classification of PD from HC	Diagnosis	UCI machine learning repository	31; 8 HC + 23 PD	Rotation forest ensemble	Accuracy = 96.93%	2012	Ozcift, 2012
					Kappa = 0.92		
					AUC = 0.97		
Classification of PD from HC	Diagnosis	UCI machine learning repository	31; 8 HC + 23 PD	SVM-RBF with 10-fold cross validation or a train-test ratio of 50:50	10-fold cross validation:	2016	Peker, 2016
					Accuracy = 98.95%		
					Sensitivity = 96.12%		
					Specificity = 100%		
					F-measure = 0.9795		
					Kappa = 0.9735		
					AUC = 0.9808		
Classification of PD from HC	Diagnosis	UCI machine learning repository	31; 8 HC + 23 PD	ELM with 10-fold cross validation	Accuracy = 88.72%	2016	Shahsavari et al., 2016
					Recall = 94.33%		
					Precision = 90.48%		
					F-score = 92.36%		
Classification of PD from HC	Diagnosis	UCI machine learning repository	31; 8 HC + 23 PD	Ensemble learning with 10-fold cross validation	Accuracy = 90.6%	2019	Sheibani et al., 2019
					Sensitivity = 95.8%		
					Specificity = 75%		
Classification of PD from HC	Diagnosis	UCI machine learning repository	31; 8 HC + 23 PD	GLRA, SVM, bagging ensemble with 5-fold cross validation	Bagging:	2017	Wu et al., 2017
					Sensitivity = 0.9796		
					Specificity = 0.6875		
					MCC = 0.6977		
					AUC = 0.9558		
					SVM:		
					Sensitivity = 0.9252		
					specificity = 0.8542		
					MCC = 0.7592		
					AUC = 0.9349		
Classification of PD from HC	Diagnosis	UCI machine learning repository	31; 8 HC + 23 PD	Decision tree classifier, logistic regression, SVM with 10-fold cross validation	SVM:	2011	Yadav et al., 2011
					Accuracy = 0.76		
					Sensitivity = 0.9745		
					Specificity = 0.13		
Classification of PD from HC	Diagnosis	UCI machine learning repository	80; 40 HC + 40 PD	KNN, SVM with 10-fold cross validation	SVM:	2019	Yaman et al., 2020
					Accuracy = 91.25%		
					Precision = 0.9125		
					Recall = 0.9125		
					F-Measure = 0.9125		
Classification of PD from HC	Diagnosis	UCI machine learning repository	31; 8 HC + 23 PD	MAP, SVM-RBF, FLDA with 5-fold cross validation	MAP:	2014	Yang et al., 2014
					Accuracy = 91.8%		
					Sensitivity = 0.986		
					Specificity = 0.708		
					AUC = 0.94		
Classification of PD from other disorders	Differential diagnosis	Collected from participants	50; 30 PD + 9 MSA + 5 FND + 1 somatization + 1 dystonia + 2 CD + 1 ET + 1 GPD	SVM, KNN, DA, naïve Bayes, classification tree with LOSO	SVM-linear:	2016	Benba et al., 2016a
					Accuracy = 90%		
					Sensitivity = 90%		
					Specificity = 90%		
					MCC = 0.794067		
					PE = 0.788177		
Classification of PD from other disorders	Differential diagnosis	Collected from participants	40; 20 PD + 9 MSA + 5 FND + 1 somatization + 1 dystonia + 2 CD + 1ET + 1 GPD	SVM (RBF, linear, polynomial, and MLP kernels) with LOSO	SVM-linear accuracy = 85%	2016	Benba et al., 2016b
Classification of PD from HC and assess the severity of PD	Diagnosis	Collected from participants	52; 9 HC + 43 PD	SVM-RBF with cross validation	Accuracy = 81.8%	2014	Frid et al., 2014
Classification of PD from HC	Diagnosis	Collected from participants	54; 27 HC + 27 PD	SVM with stratified 10-fold cross validation or leave-one-out cross validation	Accuracy = 94.4%	2018	Montaña et al., 2018
					Specificity = 100%		
					Sensitivity = 88.9%		
Classification of PD from HC	Diagnosis	Collected from participants	40; 20 HC + 20 PD	KNN, SVM-linear, SVM-RBF with leave-one-subject-out or summarized leave-one-out	SVM-linear:	2013	Sakar et al., 2013
					Accuracy = 77.50%		
					MCC = 0.5507		
					Sensitivity = 80.00%		
					Specificity = 75.00%		
Classification of PD from HC	Diagnosis	Collected from participants	78; 27 HC + 51 PD	KNN, SVM-linear, SVM-RBF, ANN, DNN with leave-one-out cross validation	SVM-RBF:	2017	Sztahó et al., 2017
					Accuracy = 84.62%		
					Precision = 88.04%		
					Recall = 78.65%		
Classification of PD from HC and assess the severity of PD	Diagnosis	Collected from participants	88; 33 HC + 55 PD	KNN, SVM-linear, SVM-RBF, ANN, DNN with leave-one-subject-out cross validation	SVM-RBF:	2019	Sztahó et al., 2019
					Accuracy = 89.3%		
					Sensitivity = 90.2%		
					Specificity = 87.9%		
Classification of PD from HC	Diagnosis	Collected from participants	43; 10 HC + 33 PD	Random forests, SVM with 10-fold cross validation and a train-test ratio of 90:10	SVM accuracy = 98.6%	2012	Tsanas et al., 2012
Classification of PD from HC	Diagnosis	Collected from participants	99; 35 HC + 64 PD	Random forest with internal out-of-bag (OOB) validation	EER = 19.27%	2017	Vaiciukynas et al., 2017
Classification of PD from HC	Diagnosis	UCI machine learning repository and participants	40 and 28; 20 HC + 20 PD and 28 PD, respectively	ELM	Training data:	2016	Agarwal et al., 2016
					Accuracy = 90.76%		
					MCC = 0.815		
					Test data:		
					Accuracy = 81.55%		
Classification of PD from HC	Diagnosis	The Neurovoz corpus	108; 56 HC + 52 PD	Siamese LSTM-based NN with 10-fold cross- validation	EER = 1.9%	2019	Bhati et al., 2019
Classification of PD from HC	Diagnosis	mPower database	2,289; 2,023 HC + 246 PD	L2-regularized logistic regression, random forest, gradient boosted decision trees with 5-fold cross validation	Gradient boosted decision trees:	2019	Tracy et al., 2019
					Recall = 0.797		
					Precision = 0.901		
					F1-score = 0.836		
Classification of PD from HC	Diagnosis	PC-GITA database	100; 50 HC + 50 PD	ResNet with train-validation ratio of 90:10	Precision = 0.92	2019	Wodzinski et al., 2019
					Recall = 0.92		
					F1-score = 0.92		
					Accuracy = 91.7%		

*ANN, artificial neural network; AUC, area under the receiver operating characteristic (ROC) curve; CART, classification and regression trees; CD, cervical dystonia; CMTNN, complementary neural network; CNN, convolutional neural network; DA, discriminant analysis; DBN, deep belief network; DNN, deep neural network; ECFA, enhanced chaos-based firefly algorithm; EFMM-OneR, enhanced fuzzy min-max neural network with the OneR attribute evaluator; ELM, extreme Learning machine; ET, extra trees or essential tremor; EWNN, evolutionary wavelet neural network; FBANN, feedforward back-propagation based artificial neural network; FKNN, fuzzy k-nearest neighbor; FLDA, Fisher's linear discriminant analysis; FND, functional neurological disorder; FNR, false negative rate; FPR, false positive rate; FURIA, fuzzy unordered rule induction algorithm; GA, genetic algorithm; GBM, gradient boosting machine; GLRA, generalized logistic regression analysis; GPD, generalized paroxysmal dystonia; GRNN, general(ized) regression neural network; HC, healthy control; HMM, hidden Markov model; IGWO-KELM, improved gray wolf optimization and kernel(-based) extreme learning machine; KELM, kernel-based extreme learning machine; KNN, k-nearest neighbors; LDA, linear discriminant analysis; LOSO, leave-one-subject-out; LS-SVM, least-square support vector machine; LSTM, long short-term memory; MAP, maximum a posteriori decision rule; MCC, Matthews correlation coefficient; MLP, multilayer perceptron; MSA, multiple system atrophy; MSE, mean squared error; NN, neural network; NNge, non-nested generalized exemplars; NPV, negative predictive value; PD, Parkinson's disease; PNN, probabilistic neural network; RBM, restricted Boltzmann machine; ResNet, residual neural network; RPART, recursive partitioning and regression trees; SCFW-KELM, subtractive clustering features weighting and kernel-based extreme learning machine; SMO, sequential minimal optimization; SVM, support vector machine; SVM-linear, support vector machine with linear kernel; SVM-RBF, support vector machine with radial basis function kernel; XGBoost, extreme gradient boosting*.

#### Movement Data (*n* = 51)

The 43 out of 51 studies using accuracy to assess model performance achieved an average accuracy of 89.1 (8.3) %, ranging from 62.1% (Prince and de Vos, 2018) to 100.0% (Surangsrirat et al., 2016; Joshi et al., 2017; Pham, 2018; Pham and Yan, 2018; Figure 4A). One study reported three machine learning methods (SVM, nearest neighbor and decision tree) achieving the highest accuracy individually (Félix et al., 2019). Out of the 51 studies, the per-study highest accuracy was achieved with SVM in 22 studies (41.5%), with ensemble learning in 13 studies (24.5%), with neural network in 9 studies (17.0%), with nearest neighbor in 4 studies (7.5%), with discriminant analysis in 1 study (1.9%), with naïve Bayes in 1 study (1.9%), and with decision tree in 1 study (1.9%). Models that do not belong to any given categories were associated with the highest per-study accuracy in two studies (3.8%; Figure 4B).

Among the 33 studies that collected movement data from recruited participants, 25 used accuracy in model evaluation, leading to an average accuracy of 87.0 (7.3) % (Table 5). The lowest and highest accuracies were 64.1% (Martínez et al., 2018) and 100.0% (Surangsrirat et al., 2016), respectively. Fifteen studies used data from the PhysioNet database (Table 5) and had an average accuracy of 94.4 (4.6) %, a lowest accuracy of 86.4% and a highest accuracy of 100%. Three studies used data from the mPower database (*n* = 2) or data sourced from another study (*n* = 1), and the average accuracy of these studies was 80.6 (16.2) %.

**Table 5:** Studies that applied machine learning models to movement data to diagnose PD (*n* = 51).

**Objectives**	**Type of diagnosis**	**Source of data**	**Number of subjects (*n*)**	**Machine learning method(s), splitting strategy and cross validation**	**Outcomes**	**Year**	**References**
Classification of PD from HC	Diagnosis	Collected from participants	103; 71 HC + 32 PD	Ensemble method of 8 models (SVM, MLP, logistic regression, random forest, NSVC, decision tree, KNN, QDA)	Sensitivity = 96% Specificity = 97% AUC = 0.98	2017	Adams, 2017
Classification of PD, HC and other neurological stance disorders	Diagnosis and differential diagnosis	Collected from participants	293; 57 HC + 27 PD + 49 AVS + 12 PNP + 48 CA + 16 DN + 25 OT + 59 PPV	Ensemble method of 7 models (logistic regression, KNN, shallow and deep ANNs, SVM, random forest, extra-randomized trees) with 90% training and 10% testing data in stratified k-fold cross-validation	8-class classification accuracy = 82.7%	2019	Ahmadi et al., 2019
Classification of PD from HC	Diagnosis	Collected from participants	137; 38 HC + 99 PD	SVM with leave-one-out-cross validation	PD vs. HC accuracy = 92.3%	2016	Bernad-Elazari et al., 2016
					Mild vs. severe accuracy = 89.8%		
					Mild vs. HC accuracy = 85.9%		
Classification of PD from HC	Diagnosis	Collected from participants	30; 14 HC + 16 PD	SVM (linear, quadratic, cubic, Gaussian kernels), ANN, with 5-fold cross-validation	Classification with ANN:	2019	Buongiorno et al., 2019
					Accuracy = 89.4%		
					Sensitivity = 87.0%		
					Specificity = 91.8%		
					Severity assessment with ANN:		
					Accuracy = 95.0%		
					sensitivity = 90.0%		
					Specificity = 99.0%		
Classification of PD from HC	Diagnosis	Collected from participants	28; 12 HC + 16 PD	NN with a train-validation-test ratio of 70:15:15, SVM with leave-one-out cross-validation, logistic regression with 10-fold cross validation	SVM: Accuracy = 85.71% Sensitivity = 83.5% Specificity = 87.5%	2017	Butt et al., 2017
Classification of PD from HC	Diagnosis	Collected from participants	28; 12 HC + 16 PD	Logistic regression, naïve Bayes, SVM with 10-fold cross validation	Naïve Bayes:	2018	Butt et al., 2018
					Accuracy = 81.45%		
					Sensitivity = 76%		
					Specificity = 86.5%		
					AUC = 0.811		
Classification of PD from HC	Diagnosis	Collected from participants	54; 27 HC + 27 PD	Naïve Bayes, LDA, KNN, decision tree, SVM-linear, SVM-RBF, majority of votes with 5-fold cross validation	Majority of votes (weighted) accuracy = 96%	2018	Caramia et al., 2018
Classification of PD, HC and PD, HC, IH	Diagnosis	Collected from participants	90; 30 PD + 30 HC + 30 IH	SVM, random forest, naïve Bayes with 10-fold cross validation	Random forest:	2019	Cavallo et al., 2019
					HC vs. PD:		
					Accuracy = 0.950		
					F-measure = 0.947		
					HC + IH vs. PD:		
					Accuracy = 0.917		
					F-measure = 0.912		
					HC vs. IH vs. PD:		
					Accuracy = 0.789		
					F-measure = 0.796		
Classification of PD from HC and classification of HC, MCI, PDNOMCI, and PDMCI	Diagnosis, differential diagnosis and subtyping	Collected from participants	PD vs. HC:	Decision tree, naïve Bayes, random forest, SVM, adaptive boosting (with decision tree or random forest) with 10-fold cross validation	Adaptive boosting with decision tree:	2015	Cook et al., 2015
			75; 50 HC + 25 PD		PD vs. HC:		
					Accuracy = 0.79		
			Subtyping:		AUC = 0.82		
			52; 18 HC + 16 PDNOMCI + 9 PDMCI + 9 MCI		Subtyping (HOA vs. MCI vs. PDNOMCI vs. PDMCI):		
					Accuracy = 0.85		
					AUC = 0.96		
Classification of PD from HC	Diagnosis	Collected from participants	580; 424 HC + 156 PD	Hidden Markov models with nearest neighbor classifier with cross validation and train-test ratio of 66.6:33.3	Accuracy = 85.51%	2017	Cuzzolin et al., 2017
Classification of PD from HC	Diagnosis	Collected from participants	80; 40 HC + 40 PD	Random forest, SVM with 10-fold cross validation	SVM-RBF:	2017	Djurić-Jovičić et al., 2017
					Accuracy = 85%		
					Sensitivity = 85%		
					Specificity = 82%		
					PPV = 86%		
					NPV = 83%		
Classification of PD from HC	Diagnosis	Collected from participants	13; 5 HC + 8 PD	SVM-RBF with leave-one-out cross validation	100% HC and PD classified correctly (confusion matrix)	2014	Dror et al., 2014
Classification of PD from HC	Diagnosis	Collected from participants	75; 38 HC + 37 PD	SVM with leave-one-out cross validation	Accuracy = 85.61%	2014	Drotár et al., 2014
					Sensitivity = 85.95%		
					Specificity = 85.26%		
Classification of PD from ET	Differential diagnosis	Collected from participants	24; 13 PD + 11 ET	SVM-linear, SVM-RBF with leave-one-out cross validation	Accuracy = 83%	2016	Ghassemi et al., 2016
Classification of PD from HC	Diagnosis	Collected from participants	41; 22 HC + 19 PD	SVM, decision tree, random forest, linear regression with 10-fold and leave-one-individual out (L1O) cross validation	SVM accuracy = 0.89	2018	Klein et al., 2017
Classification of PD from HC	Diagnosis	Collected from participants	74; 33 young HC + 14 elderly HC + 27 PD	SVM with 10-fold cross validation	Sensitivity = ~90%	2017	Javed et al., 2018
Classification of PD from HC and assess the severity of PD	Diagnosis	Collected from participants	55; 20 HC + 35 PD	SVM with leave-one-out cross validation	PD diagnosis:	2016	Koçer and Oktay, 2016
					Accuracy = 89%		
					Precision = 0.91		
					Recall = 0.94		
					Severity assessment:		
					HYS 1 accuracy = 72%		
					HYS 2 accuracy = 77%		
					HYS 3 accuracy = 75%		
					HYS 4 accuracy = 33%		
Classification of PD from HC	Diagnosis	Collected from participants	45; 20 HC + 25 PD	Naïve Bayes, logistic regression, SVM, AdaBoost, C4.5, BagDT with 10-fold stratified cross-validation apart from BagDT	BagDT: Sensitivity = 82% Specificity = 90% AUC = 0.94	2015	Kostikis et al., 2015
Classification of PD from HC	Diagnosis	Collected from participants	40; 26 HC + 14 PD	Random forest with leave-one-subject-out cross-validation	Accuracy = 94.6% Sensitivity = 91.5% Specificity = 97.2%	2017	Kuhner et al., 2017
Classification of PD from HC	Diagnosis	Collected from participants	177; 70 HC + 107 PD	ESN with 10-fold cross validation	AUC = 0.852	2018	Lacy et al., 2018
Classification of PD from HC	Diagnosis	Collected from participants	39; 16 young HC + 12 elderly HC + 11 PD	LDA with leave-one-out cross validation	Multiclass classification (young HC vs. age-matched HC vs. PD):	2018	Martínez et al., 2018
					Accuracy = 64.1%		
					Sensitivity = 47.1%		
					Specificity = 77.3%		
Classification of PD from HC	Diagnosis	Collected from participants	38; 10 HC + 28 PD	SVM-Gaussian with leave-one-out cross validation	Training accuracy = 96.9%	2018	Oliveira H. M. et al., 2018
					Test accuracy = 76.6%		
Classification of PD from HC	Diagnosis	Collected from participants	30; 15 HC + 15 PD	SVM-RBF, PNN with 10-fold cross validation	SVM-RBF:	2015	Oung et al., 2015
					Accuracy = 88.80%		
					Sensitivity = 88.70%		
					Specificity = 88.15%		
					AUC = 88.48		
Classification of PD from HC	Diagnosis	Collected from participants	45; 14 HC + 31 PD	Deep-MIL-CNN with LOSO or RkF	With LOSO:	2019	Papadopoulos et al., 2019
					Precision = 0.987		
					Sensitivity = 0.9		
					specificity = 0.993		
					F1-score = 0.943		
					With RkF:		
					Precision = 0.955		
					Sensitivity = 0.828		
					Specificity = 0.979		
					F1-score = 0.897		
Classification of PD, HC and post-stroke	Diagnosis and differential diagnosis	Collected from participants	11; 3 HC + 5 PD + 3 post-stroke	MTFL with 10-fold cross validation	PD vs. HC AUC = 0.983	2017	Papavasileiou et al., 2017
Classification of PD from HC	Diagnosis	Collected from participants	182; 94 HC + 88 PD	LSTM, CNN-1D, CNN-LSTM with 5-fold cross-validation and a training-test ratio of 90:10	CNN-LSTM:	2019	Reyes et al., 2019
					Accuracy = 83.1%		
					Precision = 83.5%		
					Recall = 83.4%		
					F1-score = 81%		
					Kappa = 64%		
Classification of PD from HC	Diagnosis	Collected from participants	60; 30 HC + 30 PD	Naïve Bayes, KNN, SVM with leave-one-out cross validation	SVM:	2019	Ricci et al., 2020
					Accuracy = 95%		
					Precision = 0.951		
					AUC = 0.950		
Classification of PD, HC and IH	Diagnosis and differential diagnosis	Collected from participants	90; 30 HC + 30 PD + 30 IH	SVM-polynomial, random forest, naïve Bayes with 10-fold cross validation	HC vs. PD, naïve Bayes or random forest:	2018	Rovini et al., 2018
					Precision = 0.967		
					Recall = 0.967		
					Specificity = 0.967		
					Accuracy = 0.967		
					F-measure = 0.967		
					HC + IH vs. PD, random forest:		
					Precision = 1.000		
					Recall = 0.933		
					Specificity = 1.000		
					Accuracy = 0.978		
					F-measure = 0.966		
					Multiclass classification, random forest:		
					Precision = 0.784		
					Recall = 0.778		
					Specificity = 0.889		
					Accuracy = 0.778		
					F-measure = 0.781		
Classification of PD, HC and IH	Diagnosis and differential diagnosis	Collected from participants	45; 15 HC + 15 PD + 15 IH	SVM-polynomial, random forest with 5-fold cross validation	HC vs. PD, random forest:	2019	Rovini et al., 2019
					Precision = 1.000		
					Recall = 1.000		
					Specificity = 1.000		
					Accuracy = 1.000		
					F-measure = 1.000		
					Multiclass classification (HC vs. IH vs. PD), random forest:		
					Precision = 0.930		
					Recall = 0.911		
					Specificity = 0.956		
					Accuracy = 0.911		
					F-measure = 0.920		
Classification of PD from ET	Differential diagnosis	Collected from participants	52; 32 PD + 20 ET	SVM-linear with 10-fold cross validation	Accuracy = 1	2016	Surangsrirat et al., 2016
					Sensitivity = 1		
					Specificity = 1		
Classification of PD from HC	Diagnosis	Collected from participants	12; 10 HC + 2 PD	Naive Bayes, LogitBoost, random forest, SVM with 10-fold cross-validation	Random forest:	2017	Tahavori et al., 2017
					Accuracy = 92.29%		
					Precision = 0.99		
					Recall = 0.99		
Classification of PD from HC	Diagnosis	Collected from participants	39; 16 HC + 23 PD	SVM-RBF with 10-fold stratified cross validation	Sensitivity = 88.9%	2010	Tien et al., 2010
					Specificity = 100%		
					Precision = 100%		
					FPR = 0.0%		
Classification of PD from HC	Diagnosis	Collected from participants	60; 30 HC + 30 PD	Logistic regression, naïve Bayes, random forest, decision tree with 10-fold cross validation	Random forest:	2018	Urcuqui et al., 2018
					Accuracy = 82%		
					False negative rate = 23%		
					False positive rate = 12%		
Classification of PD from HC	Diagnosis	PhysioNet	47; 18 HC + 29 PD	SVM, KNN, random forest, decision tree	SVM with cubic kernel:	2017	Alam et al., 2017
					Accuracy = 93.6%		
					Sensitivity = 93.1%		
					Specificity = 94.1%		
Classification of PD from HC	Diagnosis	PhysioNet	34; 17 HC + 17 PD	MLP, SVM, decision tree	MLP:	2018	Alaskar and Hussain, 2018
					Accuracy = 91.18%		
					Sensitivity = 1		
					Specificity = 0.83		
					Error = 0.09		
					AUC = 0.92		
Classification of PD from HC and assess the severity of PD	Diagnosis	PhysioNet	166; 73 HC + 93 PD	1D-CNN, 2D-CNN, LSTM, decision tree, logistic regression, SVM, MLP	2D-CNN and LSTM accuracy = 96.0%	2019	Alharthi and Ozanyan, 2019
Classification of PD from HC	Diagnosis	PhysioNet	146; 60 HC + 86 PD	SVM-Gaussian with 3- or 5-fold cross validation	Accuracy = 100%, 88.88%, and 100% in three test groups	2019	Andrei et al., 2019
Classification of PD from HC	Diagnosis	PhysioNet	166; 73 HC + 93 PD	ANN, SVM, naïve Bayes with cross validation	ANN accuracy = 86.75%	2017	Baby et al., 2017
Classification of PD from HC	Diagnosis	PhysioNet	31; 16 HC + 15 PD	SVM-linear, KNN, naïve Bayes, LDA, decision tree with leave-one-out cross validation	SVM, KNN and decision tree accuracy = 96.8%	2019	Félix et al., 2019
Classification of PD from HC	Diagnosis	PhysioNet	31; 16 HC + 15 PD	SVM-linear with leave-one-out cross validation	Accuracy = 100%	2017	Joshi et al., 2017
Classification of PD from HC	Diagnosis	PhysioNet	165; 72 HC + 93 PD	KNN, CART, decision tree, random forest, naïve Bayes, SVM-polynomial, SVM-linear, K-means, GMM with leave-one-out cross validation	SVM: Accuracy = 90.32% Precision = 90.55% Recall = 90.21% F-measure = 90.38%	2019	Khoury et al., 2019
Classification of ALS, HD, PD from HC	Diagnosis	PhysioNet	64; 16 HC + 15 PD + 13 ALS + 20 HD	String grammar unsupervised possibilistic fuzzy C-medians with FKNN, with 4-fold cross validation	PD vs. HC accuracy = 96.43%	2018	Klomsae et al., 2018
Classification of PD from HC	Diagnosis	PhysioNet	166; 73 HC + 93 PD	Logistic regression, decision trees, random forest, SVM-Linear, SVM-RBF, SVM-Poly, KNN with cross validation	KNN:	2018	Mittra and Rustagi, 2018
					Accuracy = 93.08%		
					Precision = 89.58%		
					Recall = 84.31%		
					F1-score = 86.86%		
Classification of PD from HC	Diagnosis	PhysioNet	85; 43 HC + 42 PD	LS-SVM with leave-one-out, 2- or 10-fold cross validation	Leave-one-out cross validation:	2018	Pham, 2018
					AUC = 1		
					Sensitivity = 100%		
					Specificity = 100%		
					Accuracy = 100%		
					10-fold cross validation:		
					AUC = 0.89		
					Sensitivity = 85.00%		
					Specificity = 73.21%		
					Accuracy = 79.31%		
Classification of PD from HC	Diagnosis	PhysioNet	165; 72 HC + 93 PD	LS-SVM with leave-one-out, 2- or 5- or 10-fold cross validation	Accuracy = 100%	2018	Pham and Yan, 2018
					Sensitivity = 100%		
					Specificity = 100%		
					AUC = 1		
Classification of PD from HC	Diagnosis	PhysioNet	166; 73 HC + 93 PD	DCALSTM with stratified 5-fold cross validation	Sensitivity = 99.10%	2019	Xia et al., 2020
					Specificity = 99.01%		
					Accuracy = 99.07%		
Classification of HC, PD, ALS and HD	Diagnosis and differential diagnosis	PhysioNet	64; 16 HC + 15 PD + 13 ALS + 20 HD	SVM-RBF with 10-fold cross validation	PD vs. HC:	2009	Yang et al., 2009
					Accuracy = 86.43%		
					AUC = 0.92		
Classification of PD, HD, ALS and ND from HC	Diagnosis	PhysioNet	64; 16 HC + 15 PD + 13 ALS + 20 HD	Adaptive neuro-fuzzy inference system with leave-one-out cross validation	PD vs. HC:	2018	Ye et al., 2018
					Accuracy = 90.32%		
					Sensitivity = 86.67%		
					Specificity = 93.75%		
Classification of PD from HC and assess the severity of PD	Diagnosis	mPower database	50; 22 HC + 28 PD	Random forest, bagged trees, SVM, KNN with 10-fold cross validation	Random forest:	2017	Abujrida et al., 2017
					PD vs. HC accuracy = 87.03%		
					PD severity assessment accuracy = 85.8%		
Classification of PD from HC	Diagnosis	mPower database	1,815; 866 HC + 949 PD	CNN with 10-fold cross validation	Accuracy = 62.1%	2018	Prince and de Vos, 2018
					F1 score = 63.4%		
					AUC = 63.5%		
Classification of PD from HC	Diagnosis	Dataset from Fernandez et al., 2013	49; 26 HC + 23 PD	KFD-RBF, naïve Bayes, KNN, SVM-RBF, random forest with 10-fold cross validation	Random forest accuracy = 92.6%	2015	Wahid et al., 2015

*ALS, amyotrophic lateral sclerosis; ANN, artificial neural network; AUC, area under the receiver operating characteristic (ROC) curve; AVS, acute unilateral vestibulopathy; BagDT, bootstrap aggregation for a random forest of decision trees; CA, anterior lobe cerebella atrophy; CART, classification and regression trees; DCALSTM, dual-modal with each branch has a convolutional network followed by an attention-enhanced bi-directional LSTM; DN, downbeat nystagmus syndrome; ESN, echo state network; FKNN, fuzzy k-nearest neighbor; GMM, Gaussian mixture model; HC, healthy control; HD, Huntington's disease; IH, idiopathic hyposmia; KFD, kernel Fisher discriminant; KNN, k-nearest neighbors; LDA, linear discriminant analysis; LOSO, leave-one-subject-out; LS-SVM, least-squares support vector machine; LSTM, long short-term memory; MCI, mild cognitive impairment; MIL, multiple-instance learning; MLP, multilayer perceptron; MTFL, multi-task feature learning; NN, neural network; NSVC, nu-support vector classification; OT, primary orthostatic tremor; PD, Parkinson's disease; PDMCI, PD participants who met criteria for mild cognitive impairment; PDNOMCI, PD participants with no indication of mild cognitive impairment; PNN, probabilistic neural network; PNP, sensory polyneuropathy; PPV, phobic postural vertigo; QDA, quadratic discriminant analysis; RkF, repeated k-fold; SVM, support vector machine; SVM-Poly, support vector machine with polynomial kernel; SVM-RBF, support vector machine with radial basis function kernel*.

#### MRI (*n* = 36)

Average accuracy of the 32 studies that used accuracy to evaluate the performance of machine learning models was 87.5 (8.0) %. In these studies, the lowest accuracy was 70.5% (Liu L. et al., 2016) and the highest accuracy was 100.0% (Cigdem et al., 2019; Figure 4A). Out of the 36 studies, the per-study highest accuracy was obtained with SVM in 21 studies (58.3%), with neural network in 8 studies (22.2%), with discriminant analysis in 3 studies (8.3%), with regression in 2 studies (5.6%), and with ensemble learning in 1 study (2.8%). One study (2.8%) obtained the highest per-study accuracy using models that do not belong to any of the given categories (Figure 4B). In 8 of 36 studies, neural networks were directly applied to MRI data, while the remaining studies used machine learning models to learn from extracted features, e.g., cortical thickness and volume of brain regions, to diagnose PD.

Out of 17 studies that used MRI data from the PPMI database, 16 used accuracy to evaluate model performance and the average accuracy was 87.9 (8.0) %. The lowest and highest accuracies were 70.5 and 99.9%, respectively (Table 6). In 16 out of 19 studies that acquired MRI data from human participants, accuracy was used to evaluate classification performance and an average accuracy was 87.0 (8.1) % was achieved. The lowest reported accuracy was 76.2% and the highest reported accuracy was 100% (Table 6).

**Table 6:** Studies that applied machine learning models to MRI data to diagnose PD (*n* = 36).

**Objectives**	**Type of diagnosis**	**Source of data**	**Number of subjects (*n*)**	**Machine learning method(s), splitting strategy and cross validation**	**Outcomes**	**Year**	**References**
Classification of PD from MSA	Differential diagnosis	Collected from participants	150; 54 HC + 65 PD + 31 MSA	SVM with leave-one-out-cross validation	MSA vs. PD:	2019	Abos et al., 2019
					Accuracy = 0.79		
					Sensitivity = 0.71		
					Specificity = 0.86		
					MSA vs. HC:		
					Accuracy = 0.79		
					Sensitivity = 0.84		
					Specificity = 0.74		
					MSA vs. subsample of PD:		
					Accuracy = 0.84		
					Sensitivity = 0.77		
					Specificity = 0.90		
Classification of PD from MSA	Differential diagnosis	Collected from participants	151; 59 HC + 62 PD + 30 MSA	SVM with leave-one-out-cross validation	Accuracy = 77.17%	2019	Baggio et al., 2019
					Sensitivity = 83.33%		
					Specificity = 74.19%		
Classification of PD from HC	Diagnosis	Collected from participants	94; 50 HC + 44 PD	CNN with 85 subjects for training and 9 for testing	Training accuracy = 95.24%	2019	Banerjee et al., 2019
					Testing accuracy = 88.88%		
Classification of PD from HC	Diagnosis	Collected from participants	47; 26 HC + 21 PD	SVM-linear with leave-one-out cross validation	Accuracy = 93.62%	2015	Chen et al., 2015
					Sensitivity = 90.47%		
					Specificity = 96.15%		
Classification of PD from PSP	Differential diagnosis	Collected from participants	78; 57 PD + 21 PSP	SVM with leave-one-out cross validation	Accuracy = 100%	2013	Cherubini et al., 2014a
					Sensitivity = 1		
					Specificity = 1		
Classification of PD, MSA, PSP and HC	Diagnosis and differential diagnosis	Collected from participants	106; 36 HC + 35 PD + 16 MSA + 19 PSP	Elastic Net regularized logistic regression with nested 10-fold cross validation	HC vs. PD/MSA-P/PSP:	2017	Du et al., 2017
					AUC = 0.88		
					Sensitivity = 0.80		
					Specificity = 0.83		
					PPV = 0.82		
					NPV = 0.81		
					HC vs. PD:		
					AUC = 0.91		
					Sensitivity = 0.86		
					Specificity = 0.80		
					PPV = 0.82		
					NPV = 0.89		
					PD vs. MSA/PSP:		
					AUC = 0.94		
					Sensitivity = 0.86		
					Specificity = 0.87		
					PPV = 0.88		
					NPV = 0.84		
					PD vs. MSA:		
					AUC = 0.99		
					Sensitivity = 0.97		
					Specificity = 1.00		
					PPV = 1.00		
					NPV = 0.93		
					PD vs. PSP:		
					AUC = 0.99		
					Sensitivity = 0.97		
					Specificity = 1.00		
					PPV = 1.00		
					NPV = 0.94		
					MSA vs. PSP:		
					AUC = 0.98		
					Sensitivity = 0.94		
					Specificity = 1.00		
					PPV = 1.00		
					NPV = 0.93		
Classification of HC, PD, MSA and PSP	Diagnosis and differential diagnosis	Collected from participants	64; 22 HC + 21 PD + 11 MSA + 10 PSP	SVM-linear with leave-one-out cross validation	PD vs. HC:	2011	Focke et al., 2011
					Accuracy = 41.86%		
					Sensitivity = 38.10%		
					Specificity = 45.45%		
					PD vs. MSA:		
					Accuracy = 71.87%		
					Sensitivity = 36.36%		
					Specificity = 90.48%		
					PD vs. PSP:		
					Accuracy = 96.77%		
					Sensitivity = 90%		
					Specificity = 100%		
					MSA vs. PSP:		
					Accuracy = 76.19%		
					MSA vs. HC:		
					Accuracy = 78.78%		
					Sensitivity = 54.55%		
					Specificity = 90.91%		
					PSP vs. HC:		
					Accuracy = 93.75%		
					Sensitivity = 90.00%		
					Specificity = 95.45%		
Classification of PD and atypical PD	Differential diagnosis	Collected from participants	40; 17 PD + 23 atypical PD	SVM-RBF with 10-fold cross-validation	Accuracy = 97.50%	2012	Haller et al., 2012
					TPR = 0.94		
					FPR = 0.00		
					TNR = 1.00		
					FNR = 0.06		
Classification of PD and other forms of Parkinsonism	Differential diagnosis	Collected from participants	36; 16 PD + 20 other Parkinsonism	SVM-RBF with 10-fold cross validation	Accuracy = 86.92%	2012	Haller et al., 2013
					TP = 0.87		
					FP = 0.14		
					TN = 0.87		
					FN = 0.13		
Classification of HC, PD, PSP, MSA-C and MSA-P	Diagnosis and differential diagnosis	Collected from participants	464; 73 HC + 204 PD + 106 PSP + 21 MSA-C + 60 MSA-P	SVM-RBF with 10-fold cross validation	PD vs. HC:	2016	Huppertz et al., 2016
					Sensitivity = 65.2%		
					Specificity = 67.1%		
					Accuracy = 65.7%		
					PD vs. PSP:		
					Sensitivity = 82.5%		
					Specificity = 86.8%		
					Accuracy = 85.3%		
					PD vs. MSA-C:		
					Sensitivity = 76.2%		
					Specificity = 96.1%		
					Accuracy = 94.2%		
					PD vs. MSA-P:		
					Sensitivity = 86.7%		
					Specificity = 92.2%		
					Accuracy = 90.5%		
Classification of PD from HC	Diagnosis	Collected from participants	42; 21 HC + 21 PD	SVM-linear with stratified 10-fold cross validation	Accuracy = 78.33%	2017	Kamagata et al., 2017
					Precision = 85.00%		
					Recall = 81.67%		
					AUC = 85.28%		
Classification of PD, PSP, MSA-P and HC	Diagnosis and differential diagnosis	Collected from participants	419; 142 HC + 125 PD + 98 PSP + 54 MSA-P	CNN with train-validation ratio of 85:15	PD:	2019	Kiryu et al., 2019
					Sensitivity = 94.4%		
					Specificity = 97.8%		
					Accuracy = 96.8%		
					AUC = 0.995		
					PSP:		
					Sensitivity = 84.6%		
					Specificity = 96.0%		
					Accuracy = 93.7%		
					AUC = 0.982		
					MSA-P:		
					Sensitivity = 77.8%		
					Specificity = 98.1%		
					Accuracy = 95.2%		
					AUC = 0.990		
					HC:		
					Sensitivity = 100.0%		
					Specificity = 97.5%		
					Accuracy = 98.4%		
					AUC = 1.000		
Classification of PD from HC	Diagnosis	Collected from participants	65; 31 HC + 34 PD	FCP with 36 out of the 65 subjects as the training set	AUC = 0.997	2016	Liu H. et al., 2016
Classification of PD, PSP, MSA-C and MSA-P	Differential diagnosis	Collected from participants	85; 47 PD + 22 PSP + 9 MSA-C + 7 MSA-P	SVM-linear with leave-one-out cross validation	4-class classification (MSA-C vs. MSA-P vs. PSP vs. PD) accuracy = 88%	2017	Morisi et al., 2018
Classification of PD from HC	Diagnosis	Collected from participants	89; 47 HC + 42 PD	Boosted logistic regression with nested cross-validation	Accuracy = 76.2%	2019	Rubbert et al., 2019
					Sensitivity = 81%		
					Specificity = 72.7%		
Classification of PD, PSP and HC	Diagnosis and differential diagnosis	Collected from participants	84; 28 HC + 28 PSP + 28 PD	SVM-linear with leave-one-out cross validation	PD vs. HC:	2014	Salvatore et al., 2014
					Accuracy = 85.8%		
					Specificity = 86.0%		
					Sensitivity = 86.0%		
					PSP vs. HC:		
					Accuracy = 89.1%		
					Specificity = 89.1%		
					Sensitivity = 89.5%		
					PSP vs. PD:		
					Accuracy = 88.9%		
					Specificity = 88.5%		
					Sensitivity = 89.5%		
Classification of PD, APS (MSA, PSP) and HC	Diagnosis and differential diagnosis	Collected from participants	100; 35 HC + 45 PD + 20 APS	CNN-DL, CR-ML, RA-ML with 5-fold cross-validation	PD vs. HC with CNN-DL:	2019	Shinde et al., 2019
					Test accuracy = 80.0%		
					Test sensitivity = 0.86		
					Test specificity = 0.70		
					Test AUC = 0.913		
					PD vs. APS with CNN-DL:		
					Test accuracy = 85.7%		
					Test sensitivity = 1.00		
					Test specificity = 0.50		
					Test AUC = 0.911		
Classification of PD from HC	Diagnosis	Collected from participants	101; 50 HC + 51 PD	SVM-RBF with leave-one-out cross validation	Sensitivity = 92% Specificity = 87%	2017	Tang et al., 2017
Classification of PD from HC	Diagnosis	Collected from participants	85; 40 HC + 45 PD	SVM-linear with leave-one-out, 5-fold, 0.632-fold (1-1/e), 2-fold cross validation	Accuracy = 97.7%	2016	Zeng et al., 2017
Classification of PD from HC	Diagnosis	PPMI database	543; 169 HC + 374 PD	RLDA with JFSS with 10-fold cross validation	Accuracy = 81.9%	2016	Adeli et al., 2016
Classification of PD from HC	Diagnosis	PPMI database	543; 169 HC + 374 PD	RFS-LDA with 10-fold cross validation	Accuracy = 79.8%	2019	Adeli et al., 2019
Classification of PD from HC	Diagnosis	PPMI database	543; 169 HC + 374 PD	Random forest (for feature selection and clinical score); SVM with 10-fold stratified cross validation	Accuracy = 0.93	2018	Amoroso et al., 2018
					AUC = 0.97		
					Sensitivity = 0.93		
					Specificity = 0.92		
Classification of PD, HC and prodromal	Diagnosis	PPMI database	906; 203 HC + 66 prodromal + 637 PD	MLP, XgBoost, random forest, SVM with 5-fold cross validation	MLP:	2020	Chakraborty et al., 2020
					Accuracy = 95.3%		
					Recall = 95.41%		
					Precision = 97.28%		
					F1-score = 94%		
Classification of PD from HC	Diagnosis	PPMI database	Dataset 1: 15; 6 HC + 9 PD	SVM with leave-one-out cross validation	Dataset 1:	2014	Chen et al., 2014
					EER = 87%		
			Dataset 2: 39; 21 HC + 18 PD		Accuracy = 80%		
					AUC = 0.907		
					Dataset 2:		
					EER = 73%		
					Accuracy = 68%		
					AUC = 0.780		
Classification of PD from HC	Diagnosis	PPMI database	80; 40 HC + 40 PD	Naïve Bayes, SVM-RBF with 10-fold cross validation	SVM:	2019	Cigdem et al., 2019
					Accuracy = 87.50%		
					Sensitivity = 85.00%		
					Specificity = 90.00%		
					AUC = 90.00%		
Classification of PD from HC	Diagnosis	PPMI database	37; 18 HC + 19 PD	SVM-linear with leave-one-out cross validation	Accuracy = 94.59%	2017	Kazeminejad et al., 2017
Classification of PD, HC and SWEDD	Diagnosis and subtyping	PPMI database	238; 62 HC + 142 PD + 34 SWEDD	Joint learning with 10-fold cross validation	HC vs. PD:	2018	Lei et al., 2019
					Accuracy = 91.12%		
					AUC = 94.88%		
					HC vs. SWEDD:		
					Accuracy = 94.89%		
					AUC = 97.80%		
					PD vs. SWEDD:		
					accuracy = 92.12%		
					AUC = 93.82%		
Classification of PD and SWEDD from HC	Diagnosis	PPMI database	Baseline: 238; 62 HC + 142 PD + 34 SWEDD12 months: 186; 54 HC + 123 PD + 9 SWEDD 24 months: 127; 7 HC + 88 PD + 22 SWEDD	SSAE with 10-fold cross validation	HC vs. PD: Accuracy = 85.24%, 88.14%, and 96.19% for baseline, 12 m, and 24 mHC vs. SWEDD: Accuracy = 89.67%, 95.24%, and 93.10% for baseline, 12 m, and 24 m	2019	Li et al., 2019
Classification of PD from HC	Diagnosis	PPMI database	112; 56 HC + 56 PD	RLDA with 8-fold cross validation	Accuracy = 70.5%	2016	Liu L. et al., 2016
					AUC = 71.1		
Classification of PD from HC	Diagnosis	PPMI database	60; 30 HC + 30 PD	SVM, ELM with train-test ratio of 80:20	ELM:	2016	Pahuja and Nagabhushan, 2016
					Training accuracy = 94.87%		
					Testing accuracy = 90.97%		
					Sensitivity = 0.9245		
					Specificity = 0.9730		
Classification of PD from HC	Diagnosis	PPMI database	172; 103 HC + 69 PD	Multi-kernel SVM with 10-fold cross validation		2017	Peng et al., 2017
					Accuracy = 85.78%		
					Specificity = 87.79%		
					Sensitivity = 87.64%		
					AUC = 0.8363		
Classification of PD from HC	Diagnosis and subtyping	PPMI database	109; 32 HC + 77 PD (55 PD-NC + 22 PD-MCI)	SVM with 2-fold cross validation	PD vs. HC:	2016	Peng et al., 2016
					Accuracy = 92.35%		
					Sensitivity = 0.9035		
					Specificity = 0.9431		
					AUC = 0.9744		
					PD-MCI vs. HC:		
					Accuracy = 83.91%		
					Sensitivity = 0.8355		
					Specificity = 0.8587		
					AUC = 0.9184		
					PD-MCI vs. PD-NC:		
					Accuracy = 80.84%		
					Sensitivity = 0.7705		
					Specificity = 0.8457		
					AUC = 0.8677		
Classification of PD, HC and SWEDD	Diagnosis and subtyping	PPMI database	831; 245 HC + 518 PD + 68 SWEDD	LSSVM-RBF with cross validation	Accuracy = 99.9% Specificity = 100% Sensitivity = 99.4%	2015	Singh and Samavedham, 2015
Classification of PD, HC and SWEDD	Diagnosis and differential diagnosis	PPMI database	741; 262 HC + 408 PD + 71 SWEDD	LSSVM-RBF with 10-fold cross validation	PD vs. HC accuracy = 95.37%	2018	Singh et al., 2018
					PD vs. SWEDD accuracy = 96.04%		
					SWEDD vs. HC accuracy = 93.03%		
Classification of PD from HC	Diagnosis	PPMI database	408; 204 HC + 204 PD	CNN (VGG and ResNet)	ResNet50 accuracy = 88.6%	2019	Yagis et al., 2019
Classification of PD from HC	Diagnosis	PPMI database	754; 158 HC + 596 PD	FCN, GCN with 5-fold cross validation	AUC = 95.37%	2018	Zhang et al., 2018

*APS, atypical parkinsonian syndromes; AUC, area under the receiver operating characteristic (ROC) curve; CNN, convolutional neural network; CNN-DL, convolutional neural network with discriminative localization; CR-ML, contrast ratio classifier; EER, equal error rate; ELM, extreme learning machine; FCN, fully connected network; FCP, folded concave penalized (learning); FN, false negative; FNR, false negative rate; FP, false positive; FPR, false positive rate; GCN, graph convolutional network; HC, healthy control; JFSS, joint feature-sample selection; LSSVM, least-squares support vector machine; MLP, multilayer perceptron; MSA, multiple system atrophy; MSA-C, multiple system atrophy with a cerebellar syndrome; MSA-P, multiple system atrophy with a parkinsonian type; PD, Parkinson's disease; PD-MCI, PD participants who met criteria for mild cognitive impairment; PD-NC, PD participants with no indication of mild cognitive impairment; PSP, progressive supranuclear palsy; RA-ML, radiomics based classifier; ResNet, residual neural network; RFS-LDA, robust feature-sample linear discriminant analysis; RLDA, robust linear discriminant analysis; SSAE, stacked sparse auto-encoder; SVM, support vector machine; SVM-RBF, support vector machine with radial basis function kernel; SWEDD, PD with scans without evidence of dopaminergic deficit; TN, true negative; TNR, true negative rate; TP, true positive; TPR, true positive rate; XgBoost, extreme gradient boosting*.

#### Handwriting Patterns (*n* = 16)

Fifteen out of 16 studies used accuracy in model evaluation and the average accuracy was 87.0 (6.3) % (Table 7). Among these studies, the lowest accuracy was 76.44% (Ali et al., 2019b) and the highest accuracy was 99.3% (Pereira et al., 2018; Figure 4A). The highest accuracy per-study was obtained with neural network in 6 studies (37.5%), with SVM in 5 studies (31.3%), with ensemble learning in 4 studies (25.0%), and with naïve Bayes in 1 study (6.3%; Figure 4B).

**Table 7:** Studies that applied machine learning models to handwritten patterns, SPECT, PET, CSF, other data types and combinations of data to diagnose PD (*n* = 67).

**Objectives**	**Type of diagnosis**	**Source of data**	**Type of data**	**Number of subjects (*n*)**	**Machine learning method(s), splitting strategy and cross validation**	**Outcomes**	**Year**	**References**
Classification of PD from HC	Diagnosis	HandPD	Handwritten patterns	92; 18 HC + 74 PD	LDA, KNN, Gaussian naïve Bayes, decision tree, Chi2 with Adaboost with 5- or 4-fold stratified cross validation	Chi-2 with Adaboost: Accuracy = 76.44% Sensitivity = 70.94% Specificity = 81.94%	2019	Ali et al., 2019b
Classification of PD (PD + SWEDD) from HC	Diagnosis	PPMI database	More than one	388; 194 HC + 168 PD + 26 SWEDD	Ensemble method of several SVM with linear kernel with leave-one-out cross validation	Accuracy = 94.38%	2018	Castillo-Barnes et al., 2018
Classification of PD from HC	Diagnosis	PPMI database	More than one	586; 184 HC + 402 PD	MLP, BayesNet, random forest, boosted logistic regression with a train-test ratio of 70:30	Boosted logistic regression: Accuracy = 97.159% AUC curve = 98.9%	2016	Challa et al., 2016
Classification of tPD from rET	Differential diagnosis	Collected from participants	More than one	30; 15 tPD + 15rET	Multi-kernel SVM with leave-one-out cross validation	Accuracy = 100%	2014	Cherubini et al., 2014b
Classfication of PD, HC and atypical PD	Diagnosis, differential diagnosis and subtyping	PPMI database and SNUH cohort	SPECT imaging data	PPMI: 701; 193 HC + 431 PD + 77 SWEDD snuh: 82 PD	CNN with train-validation ratio of 90:10	PPMI: Accuracy = 96.0% Sensitivity = 94.2% Specificity = 100% SNUH: Accuracy = 98.8% Sensitivity = 98.6% Specificity = 100%	2017	Choi et al., 2017
Classification of PD from HC	Diagnosis	Collected from participants	Other	270; 120 HC + 150 PD	Random forest	Classification error = 49.6% (rs11240569) Classification error = 44.8% (rs708727) Classification error = 49.3% (rs823156)	2019	Cibulka et al., 2019
Classification of PD from HC	Diagnosis	HandPD	Handwritten patterns	92; 18 HC + 74 PD	Naïve Bayes, OPF, SVM with cross-validation	SVM-RBF accuracy = 85.54%	2018	de Souza et al., 2018
Classification of PD from HC	Diagnosis	PPMI database	More than one	1194; 816 HC + 378 PD	BoostPark	Accuracy = 0.901 AUC-ROC = 0.977 AUC-PR = 0.947 F1-score = 0.851	2017	Dhami et al., 2017
Classification of PD and HC, and PD + SWEDD and HC	Diagnosis	PPMI database	More than one	430; 127 HC + 263 PD + 40 SWEDD	AdaBoost, SVM, naïve Bayes, decision tree, KNN, K-Means with 5-fold cross validation	PD vs. HC (adaboost): Accuracy = 0.98954704 Sensitivity = 0.97831978 Specificity = 0.99796748 PPV = 0.99723757 NPV = 0.98396794 LOR = 10.0058805 PD + SWEDD vs HC (adaboost): Accuracy = 0.9825784 Sensitivity = 0.97560976 Specificity = 0.98780488 PPV = 0.98360656 NPV = 0.98181818 LOR = 8.08332861	2016	Dinov et al., 2016
Classification of PD from HC	Diagnosis	Collected from participants	CSF	Cohort 1: 160; 80 HC + 80 PD Cohort 2: 60; 30 HC + 30 PD	Elastic Net and gradient boosted regression with 10-fold cross validation	Ensemble of 60 decision trees identified with gradient boosted model: Sensitivity = 85% Specificity = 75% PPV = 77% NPV = 83% AUC = 0.77	2018	Dos Santos et al., 2018
Classification of PD from HC	Diagnosis	Collected from participants	Handwritten patterns	75; 38 HC + 37 PD	SVM-RBF with stratified 10-fold cross-validation	Accuracy = 88.13% Sensitivity = 89.47% Specificity = 91.89%	2015	Drotár et al., 2015
Classification of PD from HC	Diagnosis	Collected from participants	Handwritten patterns	75; 38 HC + 37 PD	KNN, ensemble AdaBoost, SVM	SVM: Accuracy = 81.3% Sensitivity = 87.4% Specificity = 80.9%	2016	Drotár et al., 2016
Classification of IPD, VaP and HC	Differential diagnosis	Collected from participants	More than one	45; 15 HC + 15 IPD + 15 VaP	MLP, DBN with 10-fold cross validation	IPD + VaP vs HC with MLP: Accuracy = 95.68% Specificity = 98.08% Sensitivity = 92.44% VaP vs. IPD with DBN: Accuracy = 75.33% Specificity = 72.31% Sensitivity = 79.18%	2018	Fernandes et al., 2018
Classification of PD from HC	Diagnosis	Collected from participants	More than one	75; 15 HC + 60 PD blood: 75; 15 HC + 60 PD FDOPA PET: 58; 14 HC + 44 PD FDG PET: 67; 16 HC + 51 PD	SVM-linear, random forest with leave-one-out cross validation	SVM AUC for FDOPA + metabolomics: 0.98 SVM AUC for FDG + metabolomics: 0.91	2019	Glaab et al., 2019
Classification of PD, HC and SWEDD	Diagnosis and subtyping	PPMI database	More than one	666; 415 HC + 189 PD + 62 SWEDD	EPNN, PNN, SVM, KNN, classification tree with train-test ratio of 90:10	EPNN: PD vs SWEDD vs HC accuracy = 92.5% PD vs HC accuracy = 98.6% SWEDD vs HC accuracy = 92.0% PD vs. SWEDD accuracy = 95.3%	2015	Hirschauer et al., 2015
Classification of PD from HC and assess the severity of PD	Diagnosis	Picture Archiving and Communication System (PACS)	SPECT imaging data	202; 6 HC + 102 mild PD + 94 severe PD	Linear regression, SVM-RBF with a train-test ratio of 50:50	SVM-RBF: Sensitivity = 0.828 Specificity = 1.000 PPV = 0.837 NPV = 0.667 Accuracy = 0.832 AUC = 0.845 Kappa = 0.680	2019	Hsu et al., 2019
Classification of PD from VP	Differential diagnosis	Collected from participants	SPECT imaging data	244; 164 PD + 80 VP	Logistic regression, LDA, SVM with 10-fold cross-validation	SVM: Accuracy = 0.904 Sensitivity = 0.954 Specificity = 0.801 AUC = 0.954	2014	Huertas-Fernández et al., 2015
Classification of PD from HC	Diagnosis	Collected from participants	SPECT imaging data	208; 108 HC + 100 PD	SVM, KNN, NM with 3-fold cross validation	SVM: Sensitivity = 89.02% Specificity = 93.21% AUC = 0.9681	2012	Illan et al., 2012
Classification of PD from HC	Diagnosis	Collected from participants	Handwritten patterns	72; 15 HC + 57 PD	CNN with 10-fold cross validation or leave-one-out cross validation	Accuracy = 88.89%	2018	Khatamino et al., 2018
Classification of PD from HC	Diagnosis	Collected from participants	Other	10; 5 HC + 5 PD	SVM with leave-one-subject-out cross validation	Sensitivity = 0.90 Specificity = 0.90	2013	Kugler et al., 2013
Classification of PD from HC	Diagnosis	UCI machine learning repository	Handwritten patterns	72; 15 HC + 57 PD	SVM-linear, SVM-RBF, KNN with leave-one-subject-out cross validation	SVM-linear: Accuracy = 97.52% MCC = 0.9150 F-score = 0.9828	2019	İ et al., 2019
Classification of PD from HC	Diagnosis	Collected postmortem	CSF	105; 57 HC + 48 PD	SVM with 10-fold cross validation	Sensitivity = 65% Specificity = 79% AUC = 0.79	2013	Lewitt et al., 2013
Classification of PD from HC	Diagnosis	Collected from participants	CSF	78; 42 HC + 36 PD	Random forest and extreme gradient tree boosting with 10-fold cross validation	Extreme gradient tree boosting: Specificity = 78.6% Sensitivity = 83.3% AUC = 83.9%	2018	Maass et al., 2018
Classification of PD from HC or NPH	Diagnosis and differential diagnosis	Collected from participants	CSF	157; 68 HC + 82 PD + 7 NPH	SVM with 10-fold cross validation or leave-one-out cross validation	Cohort 1, PD vs HC: AUC = 0.76 Cohort 2, PD vs HC: AUC = 0.78 Cohort 3, PD vs HC: AUC = 0.31 Cohort 4, PD vs NPH: AUC = 0.88	2020	Maass et al., 2020
Classification of PD from HC	Diagnosis	PPMI database	More than one	550; 157 HC + 342 PD + 51 SWEDD	SVM, random forest, MLP, logistic regression, KNN with nested cross-validation	Motor features, SVM: Accuracy = 78.4% AUC = 84.7% Non-motor features, KNN: Accuracy = 82.2% AUC = 88%	2018	Mabrouk et al., 2019
Classification of PD from HC	Diagnosis	PPMI database	SPECT imaging data	642; 194 HC + 448 PD	CNN (LENET53D, ALEXNET3D) with 10-fold stratified cross-validation	ALEXNET3D: Accuracy = 94.1% AUC = 0.984	2018	Martinez-Murcia et al., 2018
Classification of PD from HC	Diagnosis	Collected from participants	Handwritten patterns	75; 10 HC + 65 PD	MLP, non-linear SVM, random forest, logistic regression with stratified 10-fold cross-validation	MLP: Accuracy = 84% Sensitivity = 75.7% Specificity = 88.9% Weighted Kappa = 0.65 AUC = 0.86	2015	Memedi et al., 2015
Classification of PD from HC	Diagnosis	Parkinson's Disease Handwriting Database (PaHaW)	Handwritten patterns	69; 36 HC + 33 PD	Random forest with stratified 7-fold cross-validation	Accuracy = 89.81% Sensitivity = 88.63% Specificity = 90.87% MCC = 0.8039	2018	Mucha et al., 2018
Classification of PD, MSA, PSP, CBS and HC	Differential diagnosis	Collected from participants	SPECT imaging data	578; 208 HC + 280 PD + 21 MSA + 41 PSP + 28 CBS	SVM with 5-fold cross-validation	Accuracy = 58.4–92.9%	2019	Nicastro et al., 2019
Classification of PD from HC	Diagnosis	Collected from participants	Handwritten patterns	30; 15 HC + 15 PD	KNN, decision tree, random forest, SVM, AdaBoost with 3-fold cross validation	Random forest accuracy = 0.91	2018	Nõmm et al., 2018
Classification of HC, AD and PD	Diagnosis and differential diagnosis	The authors' institutional oct database	Other	75; 27 HC + 28 PD + 20 AD	SVM-RBF with 2-, 5- and 10-fold cross validation	Accuracy = 87.7% HC sensitivity = 96.2% HC specificity = 88.2% PD sensitivity = 87.0% PD specificity = 100.0%	2019	Nunes et al., 2019
Classification of idiopathic PD, atypical Parkinsonian and ET	Differential diagnosis	Collected from participants	Other	85; 50 idiopathic PD + 26 atypical PD + 9 ET	SVM, random forest with leave-one-out cross validation	SVM accuracy = 100% Random forest accuracy = 98.5%	2019	Nuvoli et al., 2019
Classification of PD from HC	Diagnosis	PPMI database	SPECT imaging data	654; 209 HC + 445 PD	SVM-linear with leave-one-out cross validation	Accuracy = 97.86% Sensitivity = 97.75% Specificity = 98.09%	2015	Oliveira and Castelo-Branco, 2015
Classification of PD from HC	Diagnosis	PPMI database	SPECT imaging data	652; 209 HC + 443 PD	SVM-linear, KNN, logistic regression with leave-one-out cross validation	SVM-linear: Accuracy = 97.9% Sensitivity = 98.0% Specificity = 97.6%	2017	Oliveira F. et al., 2018
Classification of PD and non-PD (ET, drug-induced Parkinsonism)	Differential diagnosis	Collected from participants	SPECT imaging data	90; 56 PD + 34 non-PD	SVM-RBF with leave-one-out or 5-fold cross validation	Accuracy = 95.6%	2014	Palumbo et al., 2014
Classification of PD from HC	Diagnosis	Collected from participants	Handwritten patterns	55; 18 HC + 37 PD	Naïve Bayes, OPF, SVM-RBF with 10-fold cross validation	Naïve Bayes accuracy = 78.9%	2015	Pereira et al., 2015
Classification of PD from HC	Diagnosis	HandPD	Handwritten patterns	92; 18 HC + 74 PD	Naïve Bayes, OPF, SVM-RBF with cross-validation	SVM-RBF recognition rate (sensitivity) = 66.72%	2016	Pereira et al., 2016a
Classification of PD from HC	Diagnosis	Extended handpd dataset with signals extracted from a smart pen	Handwritten patterns	35; 21 HC + 14 PD	CNN with cross validation with a train:test ratio of 75:25 or 50:50	Accuracy = 87.14%	2016	Pereira et al., 2016b
Classification of PD from HC	Diagnosis	HandPD	Handwritten patterns	92; 18 HC + 74 PD	CNN, OPF, SVM, naïve Bayes with train-test split = 50:50	CNN-Cifar10 accuracy = 99.30% Early stage accuracy with CNN-ImageNet = 96.35% or 94.01% for Exam 3 or Exam 4	2018	Pereira et al., 2018
Classification of PD from HC	Diagnosis	UCI machine learning repository	More than one	Dataset 1: 40; 20 HC + 20 PD dataset 2: 77; 15 HC + 62 PD	Random forest, KNN, SVM-RBF, ensemble method with 5-fold cross validation	Ensemble method: Accuracy = 95.89% Specificity = 100% Sensitivity = 91.43%	2019	Pham et al., 2019
Classification of PD from HC	Diagnosis	PPMI database	More than one	618; 195 HC + 423 PD	SVM-linear, SVM-RBF, classification tree with a train-test ratio of 70:30	SVM-RBF, test set: Accuracy = 85.48% Sensitivity = 90.55% Specificity = 74.58% AUC = 88.22%	2014	Prashanth et al., 2014
Classification of PD from HC	Diagnosis and subtyping	PPMI database	SPECT imaging data	715; 208 HC + 427 PD + 80 SWEDD	SVM, naïve Bayes, boosted trees, random forest with 10-fold cross validation	SVM: Accuracy = 97.29% Sensitivity = 97.37% Specificity = 97.18% AUC = 99.26	2016	Prashanth et al., 2017
Classification of PD from HC	Diagnosis	PPMI database	More than one	584; 183 HC + 401 PD	Naïve Bayes, SVM-RBF, boosted trees, random forest with 10-fold cross validation	SVM: Accuracy = 96.40% Sensitivity = 97.03% Specificity = 95.01% AUC = 98.88%	2016	Prashanth et al., 2016
Classification of PD from HC	Diagnosis	PPMI database	Other	626; 180 HC + 446 PD	Logistic regression, random forests, boosted trees, SVM with cross validation	Accuracy > 95% AUC > 95% Random forests: Accuracy = 96.20–97.14% (95% CI)	2018	Prashanth and Dutta Roy, 2018
Classification of PD from HC	Diagnosis	mPower database	More than one	133 out of 1,513 with complete source data; 46 HC + 87 PD	Logistic regression, random forests, DNN, CNN, Classifier Ensemble, Multi-Source Ensemble learning with stratified 10-fold cross validation	Ensemble learning: Accuracy = 82.0% F1-score = 87.1%	2019	Prince et al., 2019
Classification of PD from HC	Diagnosis	HandPD	Handwritten patterns	35; 21 HC + 14 PD	Bidirectional Gated Recurrent Units with a train-validation-test ratio of 40:10:50 or 65:10:25	The Spiral dataset: Accuracy = 89.48% Precision = 0.848 Recall = 0.955 F1-score = 0.897 The Meander dataset: Accuracy = 92.24% Precision = 0.952 Recall = 0.883 F1-score = 0.924	2019	Ribeiro et al., 2019
Classification of PD from HC	Diagnosis	Collected from participants	Handwritten patterns	130; 39 elderly HC + 40 young HC + 39 PD + 6 PD (validation set) + 6 HC (validation set)	KNN, SVM-Gaussian, random forest with leave-one-out cross validation	SVM for PD vs young HC: Accuracy = 94.0% Sensitivity = 0.94 Specificity = 0.94 F1-score = 0.94 SVM for PD vs elderly HC: Accuracy = 89.3% Sensitivity = 0.89 Specificity = 0.89 F1-score = 0.89 Random forest for validation set: Accuracy = 83.3% Sensitivity = 0.92 Specificity = 0.93 F1-score = 0.92	2019	Rios-Urrego et al., 2019
Classification of IPD from non-IPD	Differential diagnosis	Collected from participants	PET imaging	87; 39 IPD + 48 non-IPD (24 MSA + 24 PSP)	SVM with leave-one-out cross validation	Accuracy = 78.16% Sensitivity = 69.29% Specificity = 85.42%	2015	Segovia et al., 2015
Classification of PD from HC	Diagnosis	Dataset from “Virgen de la Victoria” hospital	SPECT imaging data	189; 94 HC + 95 PD	SVM with 10-fold cross validation	Accuracy = 94.25% Sensitivity = 91.26% Specificity = 96.17%	2019	Segovia et al., 2019
Classification of PD from HC	Diagnosis	Collected from participants	Other	486; 233 HC + 205 PD + 48 NDD	SVM-linear with leave-batch-out cross validation	Validation AUC = 0.79 Test AUC = 0.74	2017	Shamir et al., 2017
Classification of PD from HC	Diagnosis	Collected from participants	PET imaging	350; 225 HC + 125 PD	GLS-DBN with a train-validation ratio of 80:20	Test dataset 1: Accuracy = 90% Sensitivity = 0.96 Specificity = 0.84 AUC = 0.9120 Test dataset 2: Accuracy = 86% Sensitivity = 0.92 Specificity = 0.80 AUC = 0.8992	2019	Shen et al., 2019
Classification of PD from HC	Diagnosis	Collected from participants	Other	33; 18 HC + 15 PD	SMMKL-linear with leave-one-out cross validation	Accuracy = 84.85% Sensitivity = 80.00% Specificity = 88.89% YI = 68.89% PPV = 85.71% NPV = 84.21% F1 score = 82.76%	2018	Shi et al., 2018
Classification of PD from HC	Diagnosis	Collected from participants	More than one	Plasma samples: 156; 76 HC + 80 PD; CSF samples: 77; 37 HC + 40 PD	PLS, random forest with 10-fold cross validation with train-test ratio of 70:30	PLS: AUC (plasma) = 0.77 AUC (CSF) = 0.90	2018	Stoessel et al., 2018
Classification of PD from HC	Diagnosis	PPMI database	SPECT imaging data	658; 210 HC + 448 PD	Logistic Lasso with 10-fold cross validation	Test errors: FP = 2.83% FN = 3.78% Net error = 3.47%	2017	Tagare et al., 2017
Classification of PD from HC	Diagnosis	PDMultiMC	handwritten patterns	42; 21 HC + 21 PD	CNN, CNN-BLSTM with stratified 3-fold cross validation	CNN: Accuracy = 83.33% Sensitivity = 85.71% Specificity = 80.95% CNN-BLSTM: Accuracy = 83.33% Sensitivity = 71.43% Specificity = 95.24%	2019	Taleb et al., 2019
Classification of PD from HC	Diagnosis	PPMI database and local database	SPECT imaging data	Local: 304; 113 Non-PDD + 191 PD PPMI: 657; 209 HC + 448 PD	SVM with stratified, nested 10-fold cross-validation	Local data: Accuracy = 0.88 to 0.92 PPMI: Accuracy = 0.95 to 0.97	2017	Taylor and Fenner, 2017
Classification of PD from HC	Diagnosis	Collected from participants	CSF	87; 43 HC + 44 PD	Logistic regression	Sensitivity = 0.797 specIFICITy = 0.800 AUC = 0.833	2017	Trezzi et al., 2017
Classification of PD from HC	Diagnosis	Collected from participants	Other	38; 24 HC + 14 PD	SVM-RFE with repeated leave-one-out bootstrap validation	Accuracy = 89.6%	2013	Tseng et al., 2013
Classification of MSA and PD	Differential diagnosis	Collected from participants	More than one	85; 25 HC + 30 PD + 30 MSA-P	NN	AUC = 0.775	2019	Tsuda et al., 2019
Classification of PD from HC	Diagnosis	Collected from participants	Other	59; 30 HC + 29 PD	Logistic regression, decision tree, extra tree	Extra tree AUC = 0.99422	2018	Vanegas et al., 2018
Classification of PD from HC	Diagnosis	Commercially sourced	Other	30; 15 HC + 15 PD	Decision tree	Cross validation score = 0.86 (male) Cross validation score = 0.63 (female)	2019	Váradi et al., 2019
Classification of PD from HC	Diagnosis	Collected from participants	More than one	84; 40 HC + 44 PD	CNN with train-validation-test ratio of 80:10:10	Accuracy = 97.6% AUC = 0.988	2018	Vásquez-Correa et al., 2019
Classification of PD and Parkinsonism	Differential diagnosis	The NTUA Parkinson Dataset	More than one	78; 55 PD + 23 Parkinsonism	MTL with DNN	Accuracy = 0.91 Precision = 0.83 Sensitivity = 1.0 Specificity = 0.83 AUC = 0.92	2018	Vlachostergiou et al., 2018
Classification of PD from HC	Diagnosis	PPMI database	More than one	534; 165 HC + 369 PD	pGTL with 10-fold cross validation	Accuracy = 97.4%	2017	Wang et al., 2017
Classification of PD from HC	Diagnosis	PPMI database	SPECT imaging data	645; 207 HC + 438 PD	CNN with train-validation-test ratio of 60:20:20	Accuracy = 0.972 Sensitivity = 0.983 Specificity = 0.962	2019	Wenzel et al., 2019
Classification of PD from HC	Diagnosis	Collected from participants	PET imaging	Cohort 1: 182; 91 HC + 91 PD Cohort 2: 48; 26 HC + 22 PD	SVM-linear, SVM-sigmoid, SVM-RBF with 5-fold cross validation	Cohort 1: Accuracy = 91.26% Sensitivity = 89.43% Specificity = 93.27% Cohort 2: Accuracy = 90.18% Sensitivity = 82.05% Specificity = 92.05%	2019	Wu et al., 2019
Classification of PD, MSA and PSP	Differential diagnosis	Collected from participants	PET imaging	920; 502 PD + 239 MSA + 179 PSP	3D residual CNN with 6-fold cross validation	Classification of PD: Sensitivity = 97.7% Specificity = 94.1% PPV = 95.5% NPV = 97.0% Classification of MSA: Sensitivity = 96.8% Specificity = 99.5% PPV = 98.7% NPV = 98.7% Classification of PSP: Sensitivity = 83.3% Specificity = 98.3% PPV = 90.0% NPV = 97.8%	2019	Zhao et al., 2019

*AD, Alzheimer's disease; AUC or AUC-ROC, area under the receiver operating characteristic (ROC) curve; AUC-PR, area under the precision-recall (PR) curve; BLSTM, bidirectional long short-term memory; CBS, corticobasal syndrome; CNN, convolutional neural network; CSF, cerebrospinal fluid; DBN, deep belief network; DNN, deep neural network; EPNN, enhanced probabilistic neural network; ET, essential tremor; FN, false negative; FP, false positive; GLS-DBN, group Lasso sparse deep belief network; HC, healthy control; IPD, idiopathic Parkinson's disease; KNN, k-nearest neighbors; LDA, linear discriminant analysis; LOR, log odds ratio; MCC, Matthews correlation coefficient; MLP, multilayer perceptron; MSA, multiple system atrophy; MSA-P, Parkinson's variant of multiple system atrophy; MTL, multi-task learning; NDD, neurodegenerative disease; NM, nearest mean; non-PDD, patients without pre-synaptic dopaminergic deficit; NPH, normal pressure hydrocephalus; NPV, negative predictive value; OPF, optimum-path forest; PD, Parkinson's disease; PET, positron emission tomography; pGTL, progressive graph-based transductive learning; PLS, partial least square; PNN, probabilistic neural network; PPV, positive predictive value; PSP, progressive supranuclear palsy; rET, essential tremor with rest tremor; SMMKL, soft margin multiple kernel learning; SPECT, single-photon emission computed tomography; SVM, support vector machine; SVM-RBF, support vector machine with radial basis function kernel; SVM-RFE, support vector machine-recursive feature elimination; SWEDD, PD with scans without evidence of dopaminergic deficit; tPD, tremor-dominant Parkinson's disease; VaP or VP, vascular Parkinsonism; YI, Youden's Index*.

#### SPECT (*n* = 14)

Average accuracy of 12 out of 14 studies that used accuracy to measure the performance of machine learning models was 94.4 (4.2) % (Table 7). The lowest reported accuracy was 83.2% (Hsu et al., 2019) and 97.9% (Oliveira F. et al., 2018; Figure 4A). SVM led to the highest per-study accuracy in 10 out of 14 studies (71.4%). The highest per-study accuracy was obtained with neural networks in 3 studies (21.4%) and with regression in 1 study (7.1%; Figure 4B).

#### PET (*n* = 4)

All 4 studies used sensitivity and specificity (Table 7) in model evaluation while 3 used accuracy. Average accuracy of the 3 studies was 85.6 (6.6) %, with a lowest accuracy of 78.16% (Segovia et al., 2015) and a highest accuracy of 90.72% (Wu et al., 2019; Figure 4A). Half of the 4 studies (50.0%) obtained the highest per-study accuracy with SVM (Segovia et al., 2015; Wu et al., 2019) and the other half (50.0%) with neural networks (Figure 4B).

#### CSF (*n* = 5)

All 5 studies used AUC, instead of accuracy, to evaluate machine learning models (Table 7). The average AUC was 0.8 (0.1), the lowest AUC was 0.6825 (Maass et al., 2020) and the highest AUC was 0.839 (Maass et al., 2018), respectively. Two studies obtained the highest per-study AUC with ensemble learning, 2 studies with SVM and 1 study with regression (Figure 4B).

#### Other Types of Data (*n* = 10)

Only 5 studies used accuracy to measure the performance of machine learning models (Table 7). An average accuracy of 91.9 (6.4) % was obtained, with a lowest accuracy of 84.85% (Shi et al., 2018) and a highest accuracy of 100% (Nuvoli et al., 2019; Figure 4A). Out of the 10 studies, 5 (50%) used SVM to achieve the per-study highest accuracy, 3 (30%) used ensemble learning, 1 (10%) used decision trees and 1 (10%) used machine learning models that do not belong to any given categories (Figure 4B).

#### Combination of More Than One Data Type (*n* = 18)

Out of the 18 studies that used more than one type of data, 15 used accuracy in model evaluation (Table 7). An average accuracy of 92.6 (6.1) % was obtained, and the lowest and highest accuracy among the 15 studies was 82.0% (Prince et al., 2019) and 100.0% (Cherubini et al., 2014b), respectively (Figure 4A). The per-study highest accuracy was achieved with ensemble learning in 6 studies (33.3%), with neural network in 5 studies (27.8%), with SVM in 4 studies (22.2%), with regression in 1 (5.6%) study and with nearest neighbor (5.6%) in 1 study. One study (5.6%) used machine learning models that do not belong to any given categories to obtain the highest per-study accuracy (Figure 4B).

## Discussion

### Principal Findings

In this review, we present results from published studies that applied machine learning to the diagnosis and differential diagnosis of PD. Since the number of included papers was relatively large, we focused on a high-level summary rather than a detailed description of methodology and direct comparison of outcomes of individual studies. We also provide an overview of sample size, data source and data type, for a more in-depth understanding of methodological differences across studies and their outcomes. Furthermore, we assessed (a) how large the participant pool/dataset was, (b) to what extent new data (i.e., unpublished, raw data acquired from locally recruited human participants) were collected and used, (c) the feasibility of machine learning and the possibility of introducing new biomarkers in the diagnosis of PD. Overall, *methodology* studies that proposed and tested novel technical approaches (e.g., machine learning and deep learning models, data acquisition devices, and feature extraction algorithms) have repetitively shown that features extracted from data modalities including voice recordings and handwritten patterns could lead to high patient-level diagnostic performance, while facilitating accessible and non-invasive data acquisition. Nevertheless, only a small number of studies further validated these technical approaches in *clinical* settings using local human participants recruited specifically for these studies, indicating a gap between model development and their clinical applications.

A per-study diagnostic accuracy above chance levels was achieved in all studies that used accuracy in model evaluation (Figure 4A). Apart from studies using CSF data that measured model performance with AUC, classification accuracy associated with 8 other data types ranged between 85.6% (PET) and 94.4% (SPECT), with an average of 89.9 (3.0) %. Therefore, although the small number of studies of some data types may not allow for a generalizable prediction of how well these data types can help us differentiate PD from HC or atypical Parkinsonian disorders, the application of machine learning to a variety of data types led to high accuracy in the diagnosis of PD. In addition, an accuracy significantly above chance levels was achieved in all machine learning models (Supplementary Table 1), while SVM, neural networks and ensemble learning were among the most popular model choices, all yielding great applicability to a variety of data modalities. In the meantime, when compared with other models, they led to the per-study highest classification accuracy in >50% of all cases (50.7, 51.9, and 52.3%, respectively; Supplementary Table 1). Despite the high diagnostic accuracy and performance reported, in a number of studies, data splitting strategies and the use of cross validation were not specified. For data modalities such as 3D MRI scans, when 2D slices are extracted from 3D volumes, multiple slices could be generated for one subject. Having data from the same subject across training, validation and tests sets can lead to a biased data split (Wen et al., 2020), causing data leakage and overestimation of model performance, thus compromising reproducibility of published results.

As previously discussed (Belić et al., 2019), although satisfactory diagnostic outcomes could be achieved, sample size in few studies was extremely small (<15 subjects). The application of some machine learning models, especially neural networks, typically rely on a large dataset. Nevertheless, collecting data from a large pool of participants remains challenging in clinical studies, and data generated are commonly of high dimensionality and small sample size (Vabalas et al., 2019). To address this challenge, one solution is to combine data from a local cohort with public repositories including PPMI, UCI machine learning repository, PhysioNet and many others, depending on the type of data that have been collected from the local cohort. Furthermore, when a great difference in group size is observed (i.e., class imbalance problem), labeling all samples after the majority class may lead to an undesired high accuracy. In this case, evaluating machine learning models with other metrics including precision, recall and F-1 score is recommended (Jeni et al., 2013).

Even though high diagnostic accuracy of PD has been achieved in clinical settings, machine learning approaches have also reached high accuracy as shown in the present study, while models including SVM and neural networks are particularly useful in (a) diagnosis of PD using data modalities that have been overlooked in clinical decision making (e.g., voice), and (b) identification of features of high relevance from these data. For example, the use of machine learning models with feature selection techniques allows for assessing the relative importance of features of a large feature space in order to select the most differentiating ones, which is conventionally challenging using manual approaches. For the discovery of novel markers allowing for non-invasive diagnostic options with relatively high accuracy, e.g., handwritten patterns, a small number of studies have been conducted, mostly using data from published databases. Given that these databases generally included handwritten patterns from a small number of diagnosed PD patients, sometimes under 15, it would be of great importance to validate the use of handwritten patterns in early diagnosis of PD in clinical studies of a larger scale. In the meantime, diagnosing PD using more than one data modality has led to promising results. Accordingly, supplying clinicians with non-motor data and machine learning approaches may support clinical decision making in patients with ambiguous symptom presentations, and/or improve diagnosis at an earlier stage.

An issue observed in many included studies was the insufficient or inaccurate description of methods or results, and some failed to provide accurate information of the number and type of subjects used (for example, methodology studies on early diagnosis of PD missing a table summarizing the characteristics of subjects, therefore it was challenging to understand the stage of PD in recruited patients), or how machine learning models were implemented, trained and tested. Infrequently, authors skipped basic information such as number of subjects and their medical conditions and referred to another publication. Although we attempted to list model hyperparameters and cross-validation strategies in the data extraction table, many included studies did not make this information available in the main text, leading to potential difficulties in replicating the results. Apart from these, rounding errors or inconsistent reporting of results also exist. Furthermore, although we treated the differentiation of PD from SWEDD as subtyping, there is ongoing controversy regarding whether it should be considered as differential diagnosis or subtyping (Lee et al., 2014; Erro et al., 2016; Chou, 2017; Kwon et al., 2018). Given these limitations, clinicians interested in adapting machine learning models or implementing diagnostic systems based on novel biomarkers are advised to interpret published results with care. Further, in this context we would like to stress the need for uniform reporting standards in studies using machine learning.

In both machine learning research and clinical settings, appropriately interpreting published results and methodologies is a necessary step toward an understanding of state-of-the-art methods. Therefore, vagueness in reporting not only compromises the interpretation of results but makes further methodological developments based on published research unnecessarily challenging. Moreover, for medical doctors interested in learning how machine learning methods could be applied in their domains, insufficient description of methods may lead to incorrect model implementation and failure of replication.

To enable efficient replication of published results, detailed descriptions of (a) model and architecture (hyperparameters, number and type of layers, layer-specific parameter settings, regularization strategies, activation functions), (b) implementation (programming language, machine learning and deep learning libraries used, model training and testing, metrics and model evaluation, validation strategy, optimization), and (c) version numbers of software/libraries used for both preprocessing and model implementation, are often desirable, as newer software versions may lead to differences in pre-processing and model implementation stages (Chepkoech et al., 2016).

Due to the use of imbalanced datasets in medical sciences, reporting model performance with a confusion matrix may give rise to a more comprehensive understanding of the model's ability to discriminate between PD and healthy controls. In the meantime, due to costs associated with acquisition of patient data, researchers often need to expand data collected from a local cohort using data sourced from publicly available databases or published studies. Nevertheless, unclear description of data acquisition and pre-processing protocols in some published studies may lead to challenges in the integration of newly acquired data and previously published data. Taken together, to facilitate early, refined diagnosis of PD and efficient application of novel machine learning approaches in a clinical setting, and to allow for improved reproducibility of studies on machine learning-based diagnosis and assessment of PD, a higher transparency in reporting data collection, pre-processing protocols, model implementation, and study outcomes is required.

### Limitations

In the present study, we have excluded research articles in languages other than English and results published in the form of conference abstracts, posters, and talks. Despite the ongoing discussion of advantages and importance of including conference abstracts in systematic reviews and reviews (Scherer and Saldanha, 2019), conference abstracts often do not report sufficient key information which is why we had to exclude them. However, this may lead to a publication and result bias. In addition, since the aim of the present review is to assess and summarize published studies on the detection and early diagnosis of PD, we noticed that few large-scale, multi-centric studies on subtyping or/and severity assessment of PD were therefore excluded. Given the current challenges in subtyping, severity assessment and prognosis of PD, a further step toward a more systematic understanding of the application of machine learning to neurodegenerative diseases would be to review these studies.

Moreover, due to the high inter-study variance in the data source and presentation of results, it was challenging to directly compare outcomes associated with each type of model across studies, as some studies failed to indicate whether model performance was evaluated using a test set, and/or results given by models that did not yield the best per-study performance. Results of published studies were discussed and summarized based on data and machine learning models used, and for data modalities such as PET (*n* = 4) or CSF (*n* = 5), the number of studies were too small despite the high total number of studies included. Therefore, it was improbable to assess the general performance of machine learning techniques when PET or CSF data are used.

## Conclusions

To the best of our knowledge, the present study is the first review which included results from all studies that applied machine learning methods to the diagnosis of PD. Here, we presented included studies in a high-level summary, providing access to information including (a) machine learning methods that have been used in the diagnosis of PD and associated outcomes, (b) types of clinical, behavioral and biometric data that could be used for rendering more accurate diagnoses, (c) potential biomarkers for assisting clinical decision making, and (d) other highly relevant information, including databases that could be used to enlarge and enrich smaller datasets. In summary, realization of machine learning-assisted diagnosis of PD yields high potential for a more systematic clinical decision-making system, while adaptation of novel biomarkers may give rise to easier access to PD diagnosis at an earlier stage. Machine learning approaches therefore have the potential to provide clinicians with additional tools to screen, detect or diagnose PD.

## Data Availability Statement

The original contributions generated for the study are included in the article/Supplementary Material, further inquiries can be directed to the corresponding author/s.

## Author Contributions

JM conceived and designed the study, collected the data, performed the analysis, and wrote the paper. CD and JF supervised the research. All authors contributed to the article and approved the submitted version.

## Conflict of Interest

The authors declare that the research was conducted in the absence of any commercial or financial relationships that could be construed as a potential conflict of interest.
