# Supplementary material for: Machine Learning for the Diagnosis of Parkinson's Disease: A Review of Literature
Source: Front Aging Neurosci. 2021 May 6;13:633752. doi: 10.3389/fnagi.2021.633752 (PMC8134676; doi:10.3389/fnagi.2021.633752)
Supplement: Supplementary file 1 [file Data_Sheet_1.docx]

Supplementary Material

# Machine learning methods and associated outcomes

***SVM (n = 130)***

SVM was used to analyze movement or gait data (n = 37), voice recordings (n = 32), MRI (n = 23), SPECT (n = 10), handwriting (n = 10), CSF (n = 2) and PET (n = 2). Other data such as EMG (Kugler et al., 2013), OCT imaging (Nunes et al., 2019), cardiac scintigraphy (Nuvoli et al., 2019), Patient Questionnaire of MDS-UPDRS (Prashanth and Dutta Roy, 2018), whole-blood gene expression profiles (Shamir et al., 2017) and eye movements during natural viewing (Tseng et al., 2013) have also been used to train SVM. Eight studies used more than one data types (Supplementary Figure 1).

In the 57 studies that did not compare SVM with other models, 48 used accuracy in model evaluation, leading to an average of 90.5 (7.6) % and a range between 74.0% (Chen et al., 2014) to 100% (Cherubini et al., 2014; Cherubini et al., 2014; Joshi et al., 2017; Pham, 2018; Pham and Yan, 2018; Surangsrirat et al., 2016). Nine studies (15.8%) evaluated the performance of SVM using other metrics. In 37/73 studies that compared SVM with other machine learning models, SVM achieved the highest performance. The average accuracy of the 33 studies that used accuracy in model evaluation was 89.8 (8.0) %, with a lowest and highest per-study accuracy of 70.0% (Ali et al., 2019) and 100.0% (Nuvoli et al., 2019; Hariharan et al., 2014), respectively.

***Neural networks (n = 62)***

Neural networks were applied to voice recordings (n = 24), movement or gait data (n = 11), MRI (n = 8), handwriting (n = 6), SPECT (n = 3), PET (n = 2). Eight studies used a combination of data types (Supplementary Figure 1). In 12 studies, multiple neural networks were tested.

In the 35 studies that applied neural networks to the diagnosis of PD without experimenting with other machine learning models, 28 studies used classification accuracy as the measure of model performance and achieved an average accuracy of 89.7 (8.5) %. The highest accuracy was 100% (Ali et al., 2019) and the lowest accuracy was 62.1% (Prince and de Vos, 2018). Eight out of 35 studies used metrics other than accuracy (Supplementary Table 1). In 14 out of 27 studies that compared the performance of neural networks with other classifiers, the highest per-study accuracy was achieved by neural networks (92.9 (5.5) %), with a lowest accuracy of 82.9% (Shinde et al., 2019) and a highest accuracy of 100% (Hariharan et al., 2014).

***Ensemble learning (n = 57)***

Ensemble learning models have been trained with movement or gait data (n = 18), voice recordings (n = 14), handwriting (n = 6), MRI (n = 2), CSF (n = 2) and SPECT (n = 1). Four studies used other types of data, such as SNPs (Cibulka et al., 2019), cardiac scintigraphy (Nuvoli et al., 2019), Patient Questionnaire of MDS-UPDRS (Prashanth and Dutta Roy, 2018) and EEG (Vanegas et al., 2018). In 10 studies, ensemble learning was applied to combinations of more than one data type (Supplementary Figure 1).

In 13 studies, ensemble learning was the only method used. Nine out of 13 studies used accuracy to measure the performance of ensemble learning models, with an average accuracy of 89.2 (6.5) %. In these studies, the lowest accuracy was 76.2% (Rubbert et al., 2019) and the highest accuracy was 96.93% (Ozcift, 2012). Ensemble learning achieved the highest per-study performance in 23 out of the 44 studies that compared ensemble learning with other machine learning models. Among 19 out of the 23 studies that used accuracy in model evaluation, the lowest and highest accuracy was 76.44% (Ali et al., 2019) and 98.61% (Dinov et al., 2016), and the average accuracy was 90.4 (6.6) %.

***Nearest Neighbors (n = 33)***

Nearest neighbors was applied to analyze voice recordings (n = 12), movement or gait data (n = 10), handwriting (n = 5), SPECT data (n = 2). In 4 studies, more than one data type was used (Supplementary Figure 1).

An average accuracy of 86.5 (9.5) % was observed in the 3 studies that have examined the performance of k-nearest neighbors algorithms without testing other machine learning models (Moharkan et al., 2017; Klomsae et al., 2018; Cuzzolin et al., 2017). In the 3 studies, the highest accuracy was 96.43% (Klomsae et al., 2018) and the lowest accuracy was 77.5% (Moharkan et al., 2017). Compared with other machine learning models, nearest neighbors displayed the highest accuracy in 5 out of 30 studies, leading to an average accuracy of 93.1 (6.3) %, with a lowest accuracy of 82.2% (Mabrouk et al., 2019) and a highest accuracy of 97.89% (Cai et al., 2018).

***Regression (n = 31)***

Regression has been applied to voice recordings (n = 10), movement or gait data (n = 7), SPECT (n = 4), MRI data (n = 2), CSF (n = 2), handwriting (n = 1), EEG data (n = 1) and Patient Questionnaire of MDS-UPDRS (n = 1). In 3 studies, more than one data type was used (Supplementary Figure 1).

Four out of 31 studies did not compare the performance of regression methods with other machine learning models (Du et al., 2017; Liu et al., 2016; Tagare et al., 2017; Trezzi et al., 2017). Of these studies, 3 have reported the AUC as the performance metric (Du et al., 2017; Liu et al., 2016; Trezzi et al., 2017), leading to an average of 0.93 (0.08) and a range between 0.833 (Trezzi et al., 2017) and 0.997 (Liu et al., 2016). Among the 27 studies that compared regression with other machine learning models, regression achieved the highest performance in 3 studies, yielding an accuracy of 70% (Ali et al., 2019) or 76.03% (Celik and Omurca, 2019), and an averaged AUC of 0.835 (Stoessel et al., 2018).

***Decision tree (n = 27)***

In the 27 studies, 11 applied decision tree to movement or gait data, 9 to voice recordings, 2 to handwriting, 1 to EEG and 1 to serum samples. In 3 studies, decision tree was applied to analyze combinations of data of more than one type (Supplementary Figure 1).

In the only study that used decision tree to classify HC and PD without evaluating other models, a cross validation score of 0.86 in male subjects and 0.63 in female subjects was achieved (Váradi et al., 2019). Decision tree achieved the highest per-study accuracy of 96.8% in 1 out of 26 studies that tested multiple machine learning models (Félix et al., 2019). However, the same accuracy was achieved with SVM or nearest neighbors.

***Naïve Bayes (n = 26)***

Naïve Bayes was applied to movement or gait data (n = 13), handwriting (n = 5), voice recordings (n = 4), MRI (n = 1), SPECT (n = 1). In 2 studies, combinations of different data types were used (Supplementary Figure 1). In all 26 studies, naïve Bayes was compared with other methods and achieved the highest performance in 2 studies, reaching an accuracy of 78.9% (Pereira et al., 2015) or 81.45% (Butt et al., 2018).

***Discriminant analysis (n = 12)***

Discriminant analysis has been applied to movement or gait data (n = 4), MRI (n = 3), voice recordings (n = 3), SPECT (n = 1) and handwriting (n = 1; Supplementary Figure 1). In the 4 studies that did not compare discriminant analysis with other models, the average accuracy was 74.1 (8.3) %, with a lowest accuracy of 64.1% (Martinez et al., 2018) and a highest accuracy of 81.9% (Adeli et al., 2016). In all 8 studies that compared discriminant analysis with other methods, the highest reported performance was achieved by other machine learning models.

***Other models (n = 24)***

A small percentage of studies (n = 24, 11.5%) used machine learning models that did not belong to any given categories (Supplementary Table 1). These models have been applied to voice recordings (n = 10), handwriting (n = 4), movement or gait data (n = 3), MRI data (n = 2), SPECT (n = 1) and TCS image (n = 1). Three studies used more than one type of data (Supplementary Figure 1). In the 24 studies, 10 used only one model, and 14 assessed performance of multiple models.

In the 10 studies that used one machine learning model, 9 measured model performance with accuracy and the average accuracy was 92.8 (6.9) %. The lowest accuracy was 79.6% (Khan et al., 2018) and the highest accuracy was 100.0% (Abiyev and Abizade, 2016; Dastjerd et al., 2019). Models that do not belong to any given categories were associated with the highest accuracy in 2 out of 14 studies that assessed multiple models (Kuresan et al., 2019; Yang et al., 2014). The highest accuracy in the two studies was 95.16% and 91.8%, respectively.

# Supplementary figures and tables

#

# Supplementary Figure 1. Number of included studies published each year since 2009 on machine learning applied to PD diagnosis. Studies published in the year 2020 were excluded.

#
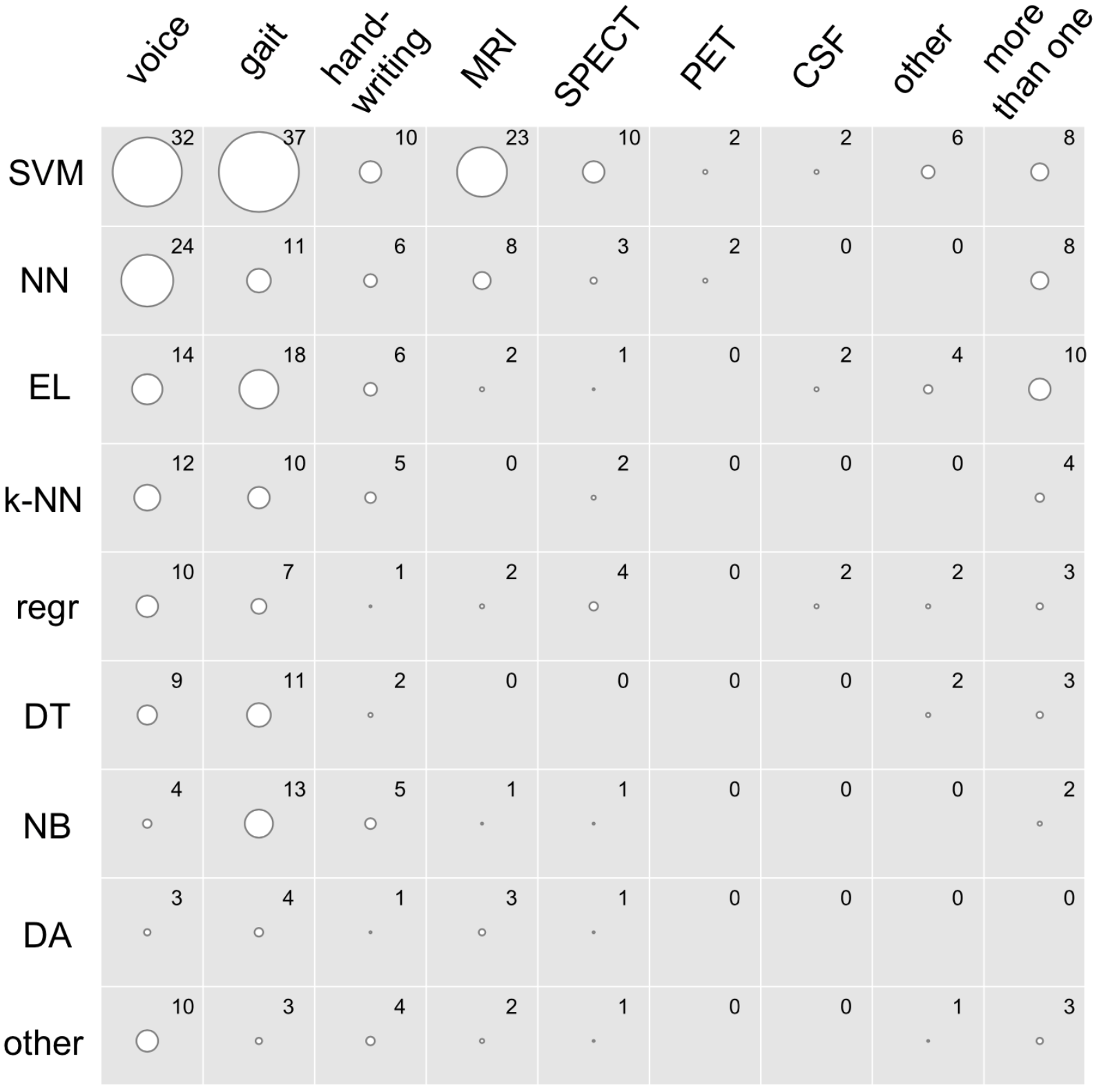


**Supplementary Figure 2.** Bubble chart showing the number of studies by the machine learning model used and the type of data analyzed. Studies using multiple machine learning models have been counted more than once. MRI: magnetic resonance imaging; SPECT: single-photon emission computed tomography; PET: positron emission tomography; CSF: cerebrospinal fluid; SVM: support vector machine; NN: neural network; EL: ensemble learning; k-NN: nearest neighbor; regr: regression; DT: decision tree; NB: naïve Bayes; DA: discriminant analysis; other: data/models that do not belong to any of the given categories.

**Supplementary Table 1**. Summary of machine learning models used in the included studies by category. SVM: support vector machine; NN: neural network; EL: ensemble learning; k-NN: nearest neighbor; DT: decision tree; NB: naïve Bayes; DA: discriminant analysis; other: data/models that do not belong to any of the given categories.

| ML model | Number of studies | Type of data | Only model tested | Performance | Compared with other models | Performance |
| --- | --- | --- | --- | --- | --- | --- |
| SVM | 130 | - Movement (37) - Voice (32) - MRI (23) - SPECT (10) - Handwriting (10) - CSF (2) - PET (2) - Other (6) - Combination (8) | In 57 studies | accuracy:  90.52 (7.65) %  min: 74%  max: 100% | In 73 studies; SVM reached highest performance in 37 studies (50.7%) | accuracy:  89.78 (8.01) %  min: 70.0%  max: 100.0% |
| NN | 62 | - Voice (24) - Movement (11) - MRI (8) - Handwriting (6) - SPECT (3) - PET (2) - Combination (8) | In 35 studies | accuracy:  89.7 (8.5) %  min: 62.1%  max: 100% | In 27 studies; NN reached highest performance in 14 studies (51.9%) | accuracy: 92.9 (5.5) %  min: 82.9%  max: 100% |
| EL | 57 | - Movement (18) - Voice (14) - Handwriting (6) - MRI (2) - CSF (2) - SPECT (1) - Other (4) - Combination (10) | In 13 studies | accuracy:  89.16 (6.47) %  min: 76.20%  max: 96.93% | In 44 studies; EL reached highest performance in 23 studies (52.3%) | accuracy: 90.56 (6.73) %  min: 76.44%  max: 98.61% |
| k-NN | 33 | - Voice (12) - Movement (10) - Handwriting (5) - SPECT (2) - Combination (4) | In 3 studies | accuracy:  86.48 (9.50) %  min: 77.50%  max: 96.43% | In 30 studies, k-NN reached highest performance in 5 studies (16.7%) | accuracy: 93.10 (6.35) %  min: 82.20%  max: 97.89% |
| regression | 31 | - Voice (10) - Movement (7) - SPECT (4) - MRI (2) - CSF (2) - Handwriting (1) - Other (2) - Combination (3) | In 4 studies | AUC:  0.93 (0.08)  min: 0.833  max: 0.997 | In 27 studies, regression reached highest performance in 3 studies (11.1%) | accuracy: 70% (Ali et al., 2019) or 76.03% (Celik and Omurca, 2019)  AUC = 0.835 (Stoessel et al., 2018) |
| DT | 27 | - Movement (11) - Voice (9) - Handwriting (2) - Other (2) - Combination (3) | In 1 study | Cross validation score = 0.86 (male) or 0.63 (female) | In 26 studies, DT reached highest performance in 1 study (3.8%) | accuracy: 96.8% |
| NB | 26 | - Movement (13) - Handwriting (5) - Voice (4) - Other (4) | In 0 study | N/A | In 26 studies, NB reached highest performance in 2 studies (7.7 %) | accuracy: 80.18 (1.80) %  min: 78.90%  max: 81.45% |
| DA | 12 | - Movement (4) - MRI (3) - Voice (3) - SPECT (1) - Handwriting (1) | In 4 studies | accuracy:  74.08 (8.29) %  min: 64.10%  max: 81.90% | In 8 studies, DA reached highest performance in 0 study (0.0%) | N/A |
| other | 24 | - Voice (10) - Handwriting (4) - Movement (3) - MRI (3) - SPECT (1) - Other (1) - Combination (2) | In 10 studies | accuracy:  92.82 (6.92) %  min: 79.60%  max: 100.00% | In 14 studies, these models reached highest performance in 2 studies (14.3%) | accuracy: 93.48 (2.38) %  min: 91.80%  max: 95.16% |
